# Cold-catalytic antitumor immunity with pyroelectric black phosphorus nanosheets†

**DOI:** 10.1039/d2sc01894b

**Published:** 2022-05-17

**Authors:** Xianbo Wu, Mei Wen, Yuyan Zou, Xinyu Gao, Chuanwan Wei, Renyu Liu, Jianghua Li, Long Wang, Xilong Li, You-Nian Liu, Wansong Chen

**Affiliations:** Hunan Provincial Key Laboratory of Micro & Nano Materials Interface Science, College of Chemistry and Chemical Engineering, Central South University Changsha Hunan 410083 China chenws@csu.edu.cn; School of Chemistry and Chemical Engineering, University of South China Hengyang Hunan 421001 China; Xiangya Hospital, Central South University Changsha Hunan 410083 China; Hefei National Laboratory for Physical Science at Microscale, University of Science and Technology of China Hefei 230026 China

## Abstract

Catalytic nanomedicine with the innate features of catalysts brings incomparable properties to biomedicine over traditional drugs. The temperature-dependent activity of catalysts provides catalytic nanomedicines with a facile strategy to control their therapeutic performance. Tuning catalytic nanomedicine by cold treatment (4–37 °C) is safe and desired for practical applications, but there is a lack of cold-catalytic platforms. Herein, with black phosphorus (BP) as a model pyroelectric nanocatalyst, we explored the potential of cold-catalysts for antitumor therapy. BP nanosheets with pyro-catalytic activity catalyze the generation of oxidative stress to activate antitumor immunity under cold treatment. Due to the cold-catalytic immunomodulation, immune memory was successfully achieved to prevent tumor metastasis and recurrence. Considering the safety and conductive depth (>10 mm) of cold in the body, pyroelectric nanocatalysts open up exciting opportunities for the development of cold-catalytic nanomedicine.

## Abstract 2

Cold-catalytic nanomedicine and the concept of cold-catalytic immunotherapy are explored with black phosphorus as a model cold catalyst.

## Graphical abstract

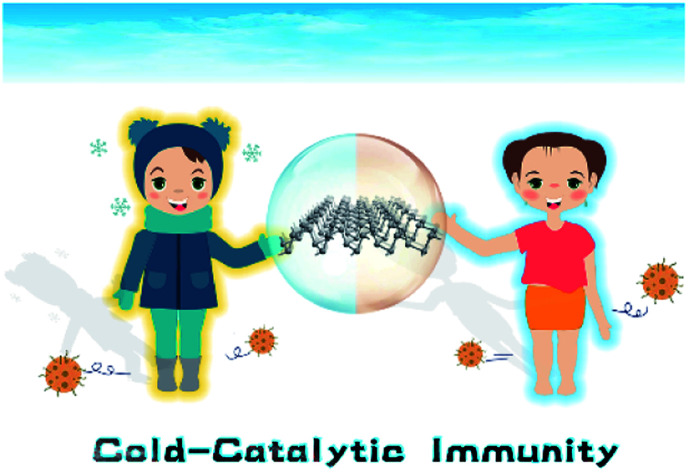

## Introduction

As the latest topic in biomedicine, catalytic nanomedicine has attracted increasing research interest because of its unique merits. The high catalytic activity of nanocatalysts is beneficial to achieving superior therapeutic performance with a minimal dosage.^1,2^ More interestingly, our previous work disclosed the promising potential of catalytic nanomedicine for immunomodulation.^3,4^ With catalytic nanomedicine as immunomodulating agents, catalytic immunotherapy could be conducted for serious diseases, including cancer, diabetes, viral infection, *etc.* Especially for cancer treatment, efficient immunomodulation is critical for boosting systematic immunity for defense against tumor metastasis and recurrence. As a unique character of catalytic nanomedicines, their therapeutic activity was highly correlated with temperature because of the temperature-dependent catalytic kinetics.^5,6^ Due to the facile operation and spatio-temporal tunability, temperature has been employed as an external stimulus for disease therapy in the clinic. So far, temperature-related catalytic nanomedicine mainly focuses on hyperthermia (>37 °C), such as hyperthermic perfusion therapy, photothermal therapy or magnetic hyperthermia therapy.^7^ Since both normal cells and cancer cells are vulnerable to necrosis under hyperthermia conditions, the safety temperature window for hyperthermia therapy is very narrow (37–42 °C).^8,9^ Comparatively, human cells are tolerable to cold (4–37 °C), making the safety temperature window for cold-catalytic therapy much wider. However, cold-catalytic therapy has always been ignored due to the lack of therapeutic platforms in response to cold conditions.

Very recently, pyroelectric materials with thermal-electricity conversion ability have been found with high sensitivity to temperature variations as small as 6 × 10^−6^ °C.^10^ When temperature varies, an internal pyroelectric field is formed, which drives the electron–hole separation for redox reactions with surrounding substrates.^6,11–15^ For example, black phosphorus (BP) nanosheets with pyroelectricity have been employed for pyro-catalytic dye decomposition and hydrogen generation under thermal cycling (15–65 °C).^16^ Intriguingly, pyroelectric materials can also be activated under cold treatment, thereby allowing them to act as cold catalysts. Inspired by the above merits, we believe that pyroelectric nanomaterials would be a promising candidate for catalytic biomedicine, especially for cold-catalytic immunomodulation. To the best of our knowledge, cold-catalytic nanomedicine and the concept of cold-catalytic immunotherapy have been rarely explored.

**Scheme 1:**
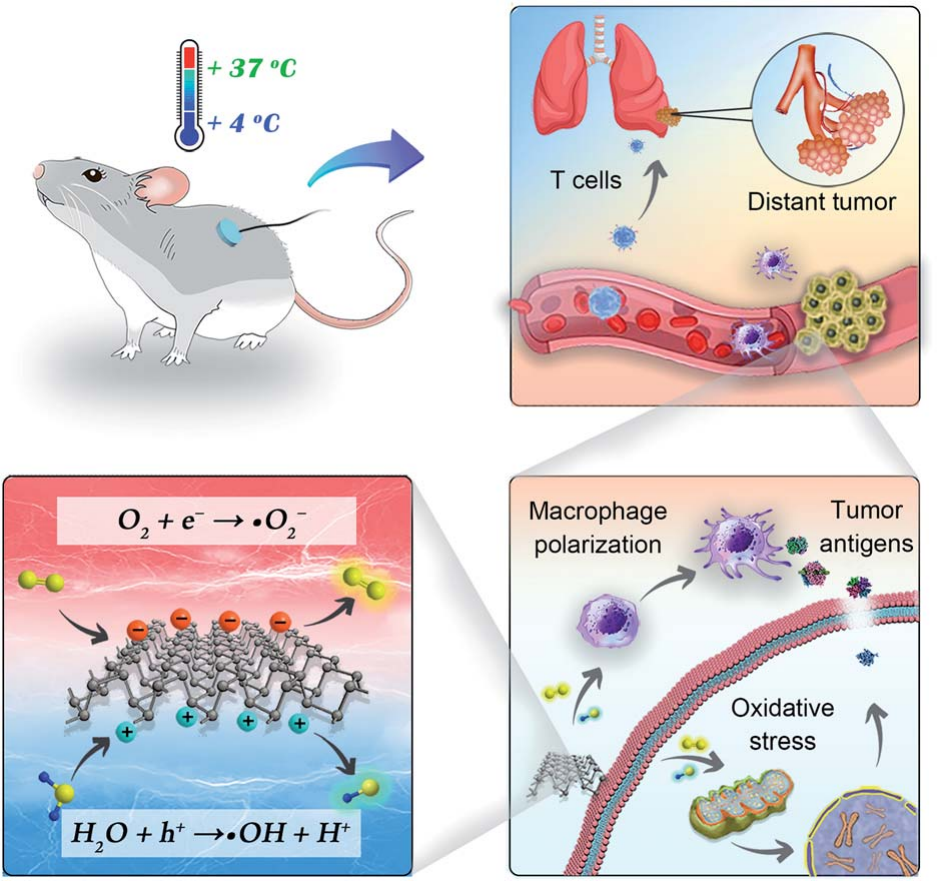
Illustration of cold-catalytic antitumor immunity with pyroelectric black phosphorus nanosheets.

In this work, we present cold-catalytic immunotherapy with black phosphorus (BP) nanosheets as an example (Scheme 1). Under the cold treatment (4–37 °C), a pyroelectric field was formed on BP nanosheets to catalytically generate reactive oxygen species (ROS). When subjected to a high level of oxidative stress, tumor cells underwent immunogenic death to release tumor associated antigens for *in situ* vaccination. Meanwhile, the elevated oxidative stress in tumors reversed the immunosuppressive tumor microenvironment. With the cold-catalytic immunotherapy of BP nanosheets, systematic antitumor immunity and the immune memory effect were successfully fabricated to eradicate tumor metastasis and recurrence. The cold temperature could be facilely controlled with a conductive depth of up to 10 mm. During the cold treatment, tissues retained integrated histological structures with minimal cold-induced injury. In addition, BP nanosheets showed good biocompatibility and biodegradability, guaranteeing their biosafety for practical applications.

## Results and discussion

**Fig. 1:**
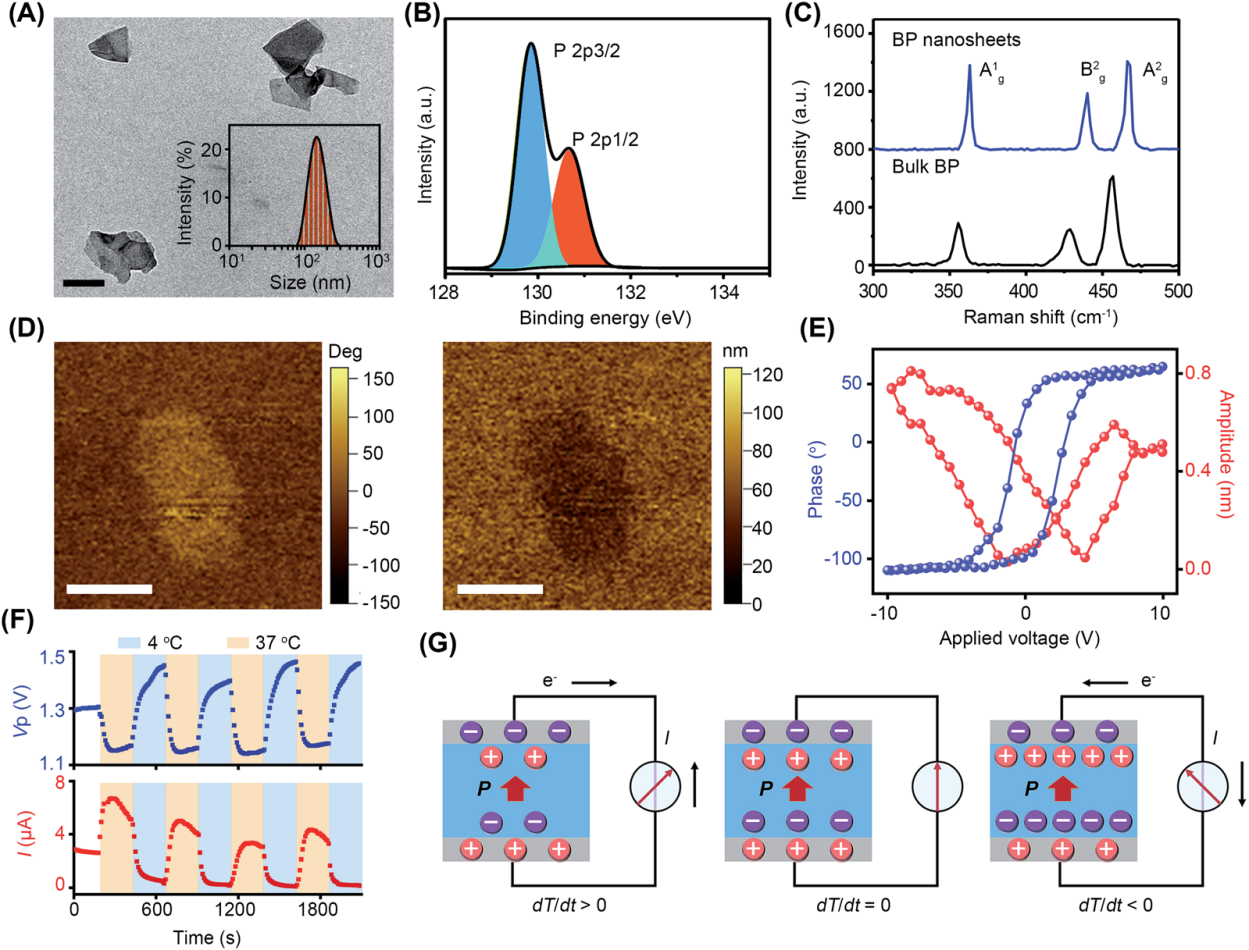
Characterization of BP nanosheets. (A) TEM images (inset: size distribution). Scale bar = 100 nm. (B) XPS spectra of BP nanosheets. (C) Raman spectrum of BP nanosheets and bulk BP crystals. (D) The out-of-plane piezoresponse force microscopy (PFM) image of BP nanosheet: the phase image (left) and amplitude image (right). Scale bar = 100 nm. (E) Piezoelectric phase and amplitude hysteresis loop of BP nanosheets. (F) Pyroelectric currents and pyroelectric potentials of BP nanosheets under temperature variations. (G) Schematic illustration of the pyroelectric effect of BP nanosheets under temperature variations.

BP nanosheets were synthesized *via* a liquid exfoliation technique following our previously reported protocols.^16,17^ The transmission electron microscopy (TEM) result reveals their free-standing nanosheet morphology (Fig. 1A). The thickness of BP nanosheets was 5 nm, according to the atomic force microscopy (AFM) measurement (Fig. S1†). The average hydrodynamic diameter of the BP nanosheets was about 142 nm with a polydispersity index (PDI) of 0.14. From the X-ray photoelectron spectroscopy (XPS) analysis (Fig. 1B and S2†), the characteristic spin–orbit double lines can be observed at 129.85 eV (P2p_3/2_) and 130.66 eV (P2p_1/2_).^18,19^ Besides, the surface charge of BP nanosheets was determined to be −13.4 mV, endowing them with long-term dispersion stability in physiological solution (Fig. S3 and S4†).^20^ As demonstrated by the X-ray diffraction (XRD) pattern, BP nanosheets retained integrated crystal structures after exfoliation (Fig. S5†). The Raman spectrum of the BP nanosheets shows three characteristic peaks A_g_^1^ (362.88 cm^−1^), B_g_^2^ (440.08 cm^−1^), and A_g_^2^ (466.58 cm^−1^), which are slightly red-shifted in comparison to those of bulk BP crystals, confirming the few layered structure of BP nanosheets (Fig. 1C).^20^

As a subset of piezoelectric materials, pyroelectric materials usually present piezoelectric effects, which are characterized by the local piezoelectric hysteresis loops.^11,20,21^ Both the vertical piezoresponse amplitude and phase diagram exhibited remarkable differences from the silicon substrate, indicating the piezoelectricity of BP nanosheets (Fig. 1D). In the voltage range of −10 V to +10 V, a typical butterfly amplitude curve was clearly observed (Fig. 1E), suggesting the constantly changing strain in the varied electric field. The phase diagram reveals a phase switch of ∼180° in the local piezoelectric hysteresis circuit of BP nanosheets. To further characterize the pyroelectricity, pyroelectric current (*I*) and voltage (*V*_p_) were measured to verify the generation of pyroelectric charge on BP nanosheets. As shown in Fig. 1F, both pyroelectric current and voltage periodically changed in a temperature-dependent manner, suggesting the pyroelectricity of BP nanosheets. A schematic diagram of the pyroelectric effect reflects the relationship between temperature variation and pyroelectric current (Fig. 1G). Temperature variation causes the oscillations of electric dipoles in the pyroelectric material, resulting in the polarization change of the pyroelectric field. Upon exposure to cold treatment (d*T*/d*t* < 0), spontaneous polarization of BP nanosheets was induced because of the reduced oscillation of electric dipoles, leading to the accumulation of free charges on the nanosheet surface.^11–13^ When BP nanosheets were connected with a short circuit, a pyroelectric current was generated owing to the flow of free charges. On the contrary, during the temperature recovery phase (d*T*/d*t* > 0), thermal vibrations of the electric dipoles made them lose orientation. As a result, the polarization intensity of BP nanosheets was reduced, giving rise to the desorption of free charge from the nanosheet surface. Likewise, pyroelectric current with reverse flow direction emerged in the short circuit.^13,22^ When temperature remained constant (d*T*/d*t* = 0), the screening charges reached an electrical balance with polarization charges in BP nanosheets, accompanying the disappearance of pyroelectric potential and current.

**Fig. 2:**
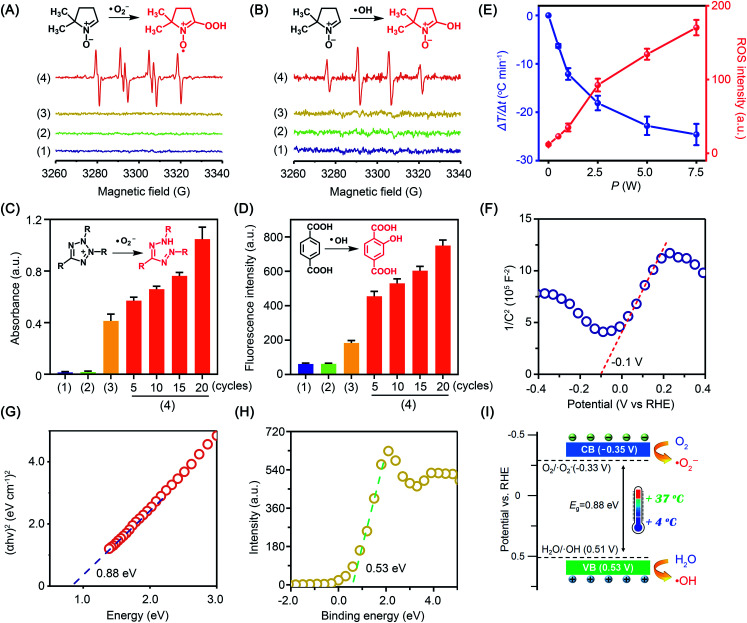
The pyroelectric catalysis ability of BP nanosheets. (A) ESR spectra of ˙O^2−^ trapped by DMPO in DMSO. (B) ESR spectra of ˙OH trapped by DMPO in water. (C) and (D) Detection of ˙OH and ˙O^2−^ in different groups using terephthalic acid and nitroblue tetrazolium as probes. All groups: (1) blank control, (2) Δ*T*, (3) BP nanosheets, and (4) BP nanosheets + Δ*T*. The temperature was varied between 4 °C and 37 °C for different cycles. (E) With a thermoelectric cooler as the cold source, the relationship of the cooler power with temperature variation and ROS generation. 2′,7′-Dichlorodihydrofluorescein was used for total ROS detection. (F) Mott–Schottky plot of BP nanosheets. (G) Tauc plot for determining the band gap of BP nanosheets. (H) XPS valence band spectrum of BP nanosheets. (I) Energy band diagrams of BP nanosheets and the ROS generating mechanism.

ROS was analyzed by electron spin resonance (ESR) spectroscopy with 5,5-dimethyl-1-pyrroline-*N*-oxide (DMPO) as a spin trapping agent.^23^ During the experiment, temperature variation was precisely controlled using a thermoelectric cooler. Obviously, the characteristic quartet signal of the DMPO–˙O_2_^−^ adduct (*A*_N_ = 13.8 G, *A*_Hβ_ = 11.8 G, *g* = 2.0068) with an intensity ratio of 1 : 2 : 2 : 1 was detected in anhydrous dimethyl sulfoxide (DMSO). When the experiment was carried out in water to quench ˙O_2_^−^, the characteristic signal of the DMPO–˙OH adduct (*A*_N_ = 14.8 G, *A*_Hβ_ = 14.8 G, *g* = 2.0064) was captured. Comparatively, little radical signal was detected for BP nanosheets without cold treatment (Fig. 2A and B). To further study the radical generation along with cold treatment, nitroblue tetrazolium and terephthalic acid were taken as fluorescent probes of ˙O_2_^−^ and ˙OH, respectively. Nitroblue tetrazolium could be oxidized by ˙O_2_^−^ to blue monoformazan with a maximum absorption wavelength of 680 nm. Nonfluorescent terephthalic acid reacts with ˙OH to generate fluorescent hydroxyl terephthalic acid. As expected, accompanying cold treatment, both ˙O_2_^−^ and ˙OH were gradually generated from BP nanosheets by virtue of the pyroelectric effect (Fig. 2C, D and S6†). Notably, ROS generation was mediated by the pyroelectric effect of BP nanosheets, which was induced by the temperature variation regardless of the cooling or heating process (Fig. S7†). When exposed to different temperature gradients, a larger temperature variation rate would provide a higher efficiency of ROS generation (Fig. S8†). The overall ROS generation was correlated with the temperature variation rate, which could be facilely controlled by the input power of the thermoelectric cooler (Fig. 2E and S6†). To disclose the mechanism underlying cold-catalytic ROS generation, we detected the energy band positions of BP nanosheets. From the Mott–Schottky curve (Fig. 2F), the flat band potential was detected to be −0.1 V *versus* the reversible hydrogen electrode (RHE). The band gap of BP nanosheets was 0.88 eV according to the Tauc plot (Fig. 2G and S9†), and the valence band (VB) was 0.53 V *vs.* RHE from the XPS valence band (VB) spectrum. Thus, the inherent VB edge is more positive than the redox potentials of H_2_O/˙OH (0.51 V),^20,24,25^ allowing H_2_O to react with the VB holes for ˙OH generation (Fig. 2H and I). Meanwhile, the conduction band (CB) of the nanosheets was calculated to be −0.35 V *vs.* RHE, which is more negative than the redox potential of O_2_/˙O_2_^−^ (−0.33 V).^26^ Electrons can transfer from the CB of BP nanosheets to surrounding O_2_ for ˙O_2_^−^ generation (Fig. 2I). Therefore, BP nanosheets were able to pyro-catalytically generate ROS from both H_2_O and O_2_ under cold treatment (eqn (1)–(3)). To further verify this mechanism, we measured the ROS generation under a N_2_ atmosphere (Fig. S10†). As expected, BP nanosheets can still produce ROS under cold treatment (Fig. S10†), although it is slightly lower than that in the normoxic environment. Therefore, the pyro-catalytic process of BP nanosheets gets rid of the high reliance on O_2_, which would benefit ROS generation under tumor hypoxia conditions.1
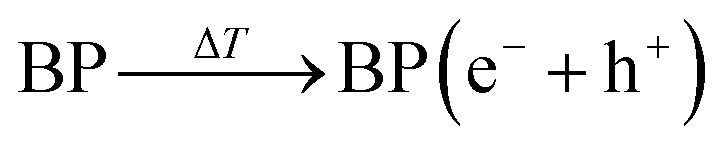

2 O_2_ + e^−^ → ˙O_2_^−^

3 H_2_O + h^+^ → ˙OH + H^+^

**Fig. 3:**
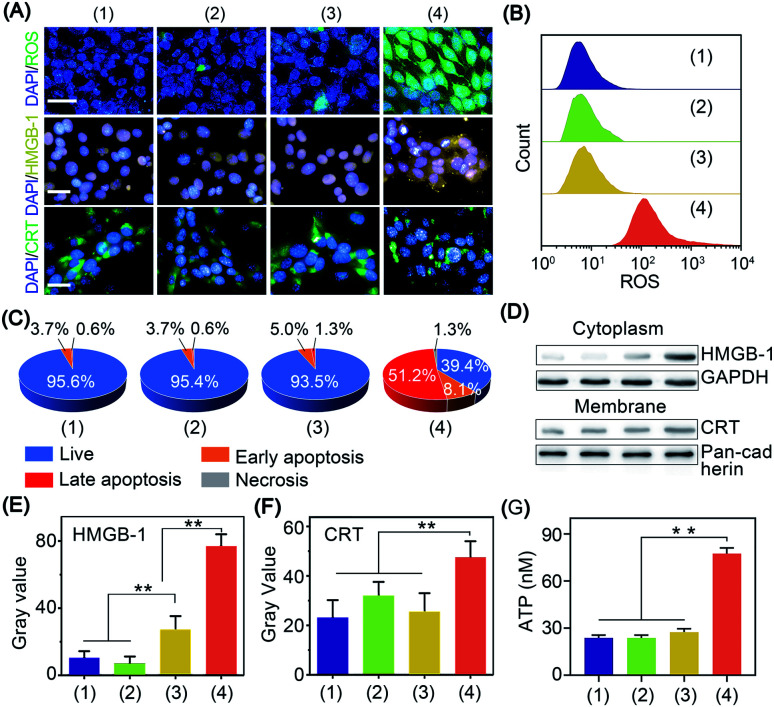
Cold-catalytic immunogenic cell death with BP nanosheets. (A) Fluorescence images of intracellular oxidative stress, CRT and HMGB-1. Nuclei were stained with DAPI with blue fluorescence (scale bars = 20 μm). (B) Intracellular fluorescence intensity of oxidative stress was analyzed by flow cytometry. (C) Quantitative results of cellular apoptosis after different treatments. (D) Western blot analysis of HMGB-1 and CRT after different treatments: (1) PBS, (2) Δ*T*, (3) BP nanosheets, and (4) Δ*T* + BP nanosheets. (E) and (F) Relative expression of cytoplasmic HMGB-1 and membrane CRT was normalized to GAPDH and pan-cadherin, respectively. (G) ATP release after different treatments. Groups: (1) PBS, (2) Δ*T*, (3) BP nanosheets, and (4) BP nanosheets + Δ*T*. **p < 0.01.

The *in vitro* cold-catalytic activity of BP nanosheets was studied in 4T1 tumor cells. BP nanosheets were labeled with fluorescent rhodamine B (RhB) and incubated with 4T1 tumor cells. The intracellular fluorescence intensity gradually increased with prolonged incubation time, verifying the internalization of BP nanosheets by tumor cells (Fig. S11†). To evaluate the cold-catalytic ROS generation, 4T1 tumor cells were incubated with BP nanosheets and then subjected to cold treatment. 2,7-Dichlorofluorescein diacetate (DCFH-DA) was used as a probe to detect intracellular oxidative stress. For cells treated with BP nanosheets or cold treatment alone, the faint intracellular fluorescence was close to that of the blank group. After the addition of BP nanosheets and cold treatment, the intracellular fluorescence intensity was sharply increased by 17.1 times (Fig. 3A and B), suggesting elevated oxidative stress. Therefore, BP nanosheets could increase intracellular oxidative stress *via* pyro-catalysis in response to cold treatment. To mimic the hypoxic condition of tumors, 4T1 cells were incubated in a medium bubbled with N_2_, and sealed with paraffin oil. Upon the cold-catalytic treatment with BP nanosheets, the intracellular fluorescence intensity was increased by 11.2 times, confirming the ROS generation in the hypoxic tumor microenvironment (Fig. S12†). Mitochondria as a critical organelle are involved in maintaining cellular redox balance. A high level of oxidative stress would destroy mitochondrial integrity and then initiate cell death. The mitochondrial dysfunction was examined using the 5,5′,6,6′-tetrachloro1-1,1′,3,3′-tetraethyl-imidacarbocyanine (JC-1) probe. The ratio of green-to-red fluorescence (G/R) was calculated and compared among different groups. Consistent with the oxidative stress staining results (Fig. S13†), the G/R ratio was sharply increased by 6.1 times in the presence of BP nanosheets with cold treatment, revealing the mitochondrial dysfunction in cold-catalytic therapy.

The antitumor efficacy of BP nanosheets under cold treatment was examined with cell apoptosis analysis and live/dead staining. Cold treatment alone showed a barely detectable influence on cell viability, and almost all the tumor cells were stained with calcein acetoxymethyl with green fluorescence (Fig. S14†). Likewise, BP nanosheets themselves without cold treatment display minimal cytotoxicity. Surprisingly, after BP nanosheet-mediated cold-catalytic therapy, nearly 60% of tumor cells were apoptotic and stained with propidium iodide with red fluorescence (Fig. 3C and S14, S15†).

*In situ* vaccination utilizes the autologous tumor-associated antigens of patients to initiate systematic antitumor immunity, thus circumventing the individual differences among patients.^27,28^ Moreover, *in situ* vaccination allows immune therapeutic agents to be locally injected into tumors to trigger the systemic antitumor immunity of the host.^27,28^ However, most tumor cells present low immunogenicity to avoid their recognition by the host immune system.^29,30^ To achieve efficient antitumor immunotherapy, tumor cells are expected to expose tumor-associated antigens *via* immunogenic cell death and stimulate subsequent antitumor immunity.^31^ Herein, the immunogenic cell death was characterized by the leakage of three biomarkers, *i.e.*, adenosine triphosphate (ATP), calreticulin (CRT), and high mobility group box 1 (HMGB-1).^32^ For cells in the blank control group, CRT and HMGB-1 were mainly distributed in the cytoplasm and nucleus of tumor cells, respectively (Fig. 3A). After cold-catalytic therapy of BP nanosheets, most of the CRT was aggregated on the plasma membrane, and HMGB-1 spread from the cell nucleus to the cytoplasm (Fig. S16†). In addition, the expression of DAMPs was quantitatively examined through western blot analysis (Fig. 3D–F). The cytoplasmic expression of HMGB-1 increased by 623.0%; meanwhile the content of membrane CRT was increased by 103.8%. Therefore, BP nanosheets with cold-catalytic activity successfully induced immunogenic cell death of tumors and released DAMPs. At the same time, ATP secretion from tumor cells was sharply increased about 3.2-fold (Fig. 3G). All these results suggest that BP nanosheets with cold-catalytic activity successfully induced immunogenic cell death of tumors.

**Fig. 4:**
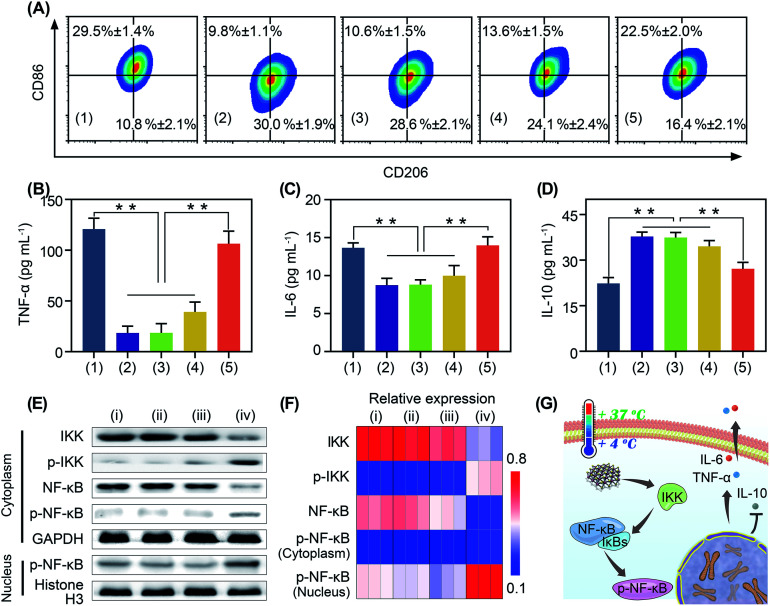
The pyroelectric catalysis mediated immunological regulations of BP nanosheets *in vitro*. (A) Flow cytometry analysis of the population of M1/M2 macrophages after different treatments. (B)–(D) Secretion of TNF-α, IL-6 and IL-10 after different treatments. Groups: (1) lipopolysaccharides, (2) IL-4, (3) IL-4 + Δ*T*, (4) IL-4 + BP nanosheets, and (5) IL-4 + BP nanosheets + Δ*T*. **p < 0.01. (E) Western blot analysis of the NF-κB signaling pathway after different treatments: (i) PBS, (ii) Δ*T*, (iii) BP nanosheets, and (iv) BP nanosheets + Δ*T*. (F) Relative expression of IKK, p-IKK, NF-κB, and cytoplasmic p-NF-kB was normalized to GAPDH. Relative expression of nuclear p-NF-κB was normalized to histone H3. (G) Mechanism of cold-catalytic macrophage polarization in the presence of BP nanosheets.

Immunotherapy is seriously impeded by the tumor immunosuppressive microenvironment because of dysfunction of immune cells, especially macrophage polarization towards tumor associated macrophages.^32,33^ Tumor associated macrophages are tightly associated with the immune resistance of tumor cells.^32,33^ Regulating the polarization of tumor associated macrophages is of great significance for anti-tumor immunotherapy.^34,35^ It has been reported that oxidative stress can stimulate macrophage repolarization from the M2 phenotype to pro-inflammatory M1 macrophages.^31,36^ Encouraged by the cold-catalytic ROS generation ability of BP nanosheets, we expected that they could promote macrophage repolarization to reverse the tumor immunosuppressive microenvironment. To verify our assumption, M2 macrophages, pre-polarized by interleukin-4 (IL-4), were incubated with BP nanosheets and then subjected to cold treatment. A bacterial endotoxin (*i.e.*, lipopolysaccharides) was employed as a positive control because of its classical role in M1 macrophage activation. Macrophage polarization markers (*i.e.*, CD86 and CD206) were analyzed after the internalization of BP nanosheets (Fig. 4A and S11, S17†). Similar to lipopolysaccharides, BP nanosheets with cold treatment significantly increased the M1 population from 9.8% to 22.5%, together with a decrease in the M2 population from 30.0% to 16.4%. In addition, the cell viability of macrophages remained over 75% during the polarization by BP nanosheets (Fig. S18†). Meanwhile, the expression of proinflammatory cytokines, including tumor necrosis factor-α (TNF-α) and IL-6, was elevated by 478.3% and 60.0%, respectively; whereas IL-10 as a typical anti-inflammatory cytokine was down-regulated by 28.1% (Fig. 4B–D). By contrast, neither cold treatment nor BP nanosheets themselves affected macrophage repolarization. To disclose the mechanism underlying macrophage repolarization, nuclear factor-κB (NF-κB) signaling as a critical pathway to mediate inflammation was investigated through western blot.^37^ Compared with the blank control group, BP nanosheets with cold treatment significantly increased the level of IKK phosphorylation by 286.9%. Moreover, the expression of NF-κB (NF-κB) was decreased by 67.0% and phosphorylated NF-κB (p-NF-κB) was increased by 103.1% in the cytoplasm, accompanied by the elevation of nuclear p-NF-κB by 73.4%. Thus, p-NF-κB was transferred from the cytoplasm to the nucleus (Fig. 4E and F), suggesting the successful activation of the NF-κB signaling pathway. Subsequently, the NF-κB signaling pathway promoted the secretion of proinflammatory cytokines (*e.g.*, TNF-α and IL-6), and blocked the expression of anti-inflammatory cytokines (*e.g.*, IL-10). Therefore, BP nanosheets with cold-catalytic activity activated the NF-κB signaling pathway of macrophages to promote macrophage repolarization (Fig. 4G), thereby reversing the immunosuppressive tumor microenvironment.

**Fig. 5:**
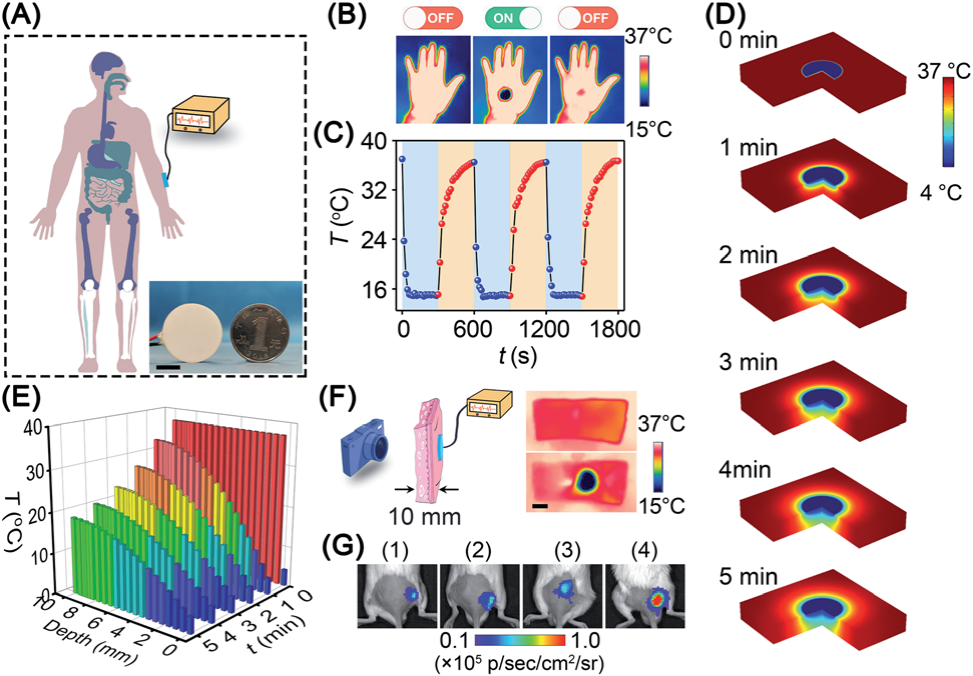
Potential of BP nanosheets for interventional therapy. (A) Schematic illustration of an interventional device for cold-catalytic therapy in the body. Inset: a thermoelectric cooler (*d* = 25 mm) was employed as the cold source (scale bar = 10 mm). (B) and (C) The temperature of the human hand was decreased using a thermoelectric cooler and then recovered spontaneously. (D) The temperature field of tissue after cold treatment for different time periods was simulated with COMSOL Multiphysics software. (E) Cold conduction in human tissue within 5 min. (F) Thermal imaging of skin tissues (thickness = 10 mm) before and after cold treatment. Scale bar = 20 mm. (G) Intratumoral oxidative stress after different treatments: (1) PBS, (2) Δ*T*, (3) BP nanosheets, and (4) BP nanosheets + Δ*T*.

Given the promising *in vitro* results above, we investigated the *in vivo* cold-catalytic activity of BP nanosheets. To induce a local cold environment *in vivo*, a thermoelectric cooler was employed as the cold source to precisely control the temperature variation (Fig. 5A). The temperature of the human hand before and after cold treatment was monitored using a thermal imaging camera (Fig. 5B and C). Obviously, upon the cold treatment with the thermoelectric cooler, the local temperature of the human hand sharply reduced to 15 °C. When the cold treatment stopped, the hand temperature gradually recovered to normal (∼36 °C), owing to the spontaneous thermoregulation of the human body. Moreover, the thermoelectric cooler can be manufactured as small as a coin (*d* = 25 mm), making it possible to work as an interventional device for *in vivo* applications. To investigate cold conduction in human tissue, the temperature field of tissue after cold treatment was simulated with COMSOL Multiphysics software (Fig. 5D). Although cold gradually decayed along with the increase in tissue depth, there remained 19 °C of temperature decrease (*i.e.*, 37 °C → 18 °C) at a tissue depth of 10 mm (Fig. 5E). Cold permeability was further confirmed *ex vivo* with pork skin (10 mm thickness). Consistent with the simulation results, the local temperature was sharply decreased to ∼16 °C at a tissue depth of 10 mm (Fig. 5F). Due to the high temperature sensitivity of pyroelectricity, a temperature variation between 37 °C and 16 °C was sufficient to trigger the pyro-catalytic ROS generation (Fig. S19†). Furthermore, cold-catalytic oxidative stress *in vivo* was detected using the chemiluminescent probe L-012 in tumor-bearing mice (Fig. 5G). For mice that received intratumoral injections of BP nanosheets only, the tumoral oxidative stress signal was almost the same as that of the blank control group. On the contrary, for mice that received cold treatment post intratumoral injection of BP nanosheets, the tumoral signal of oxidative stress was remarkably increased 8.1-fold, verifying the cold-catalytic activity of BP nanosheets *in vivo* (Fig. S20 and S21†)*.* After cold treatment, skin tissues retained integrated histological structures with minimal cold-induced injury (Fig. S22†).

**Fig. 6:**
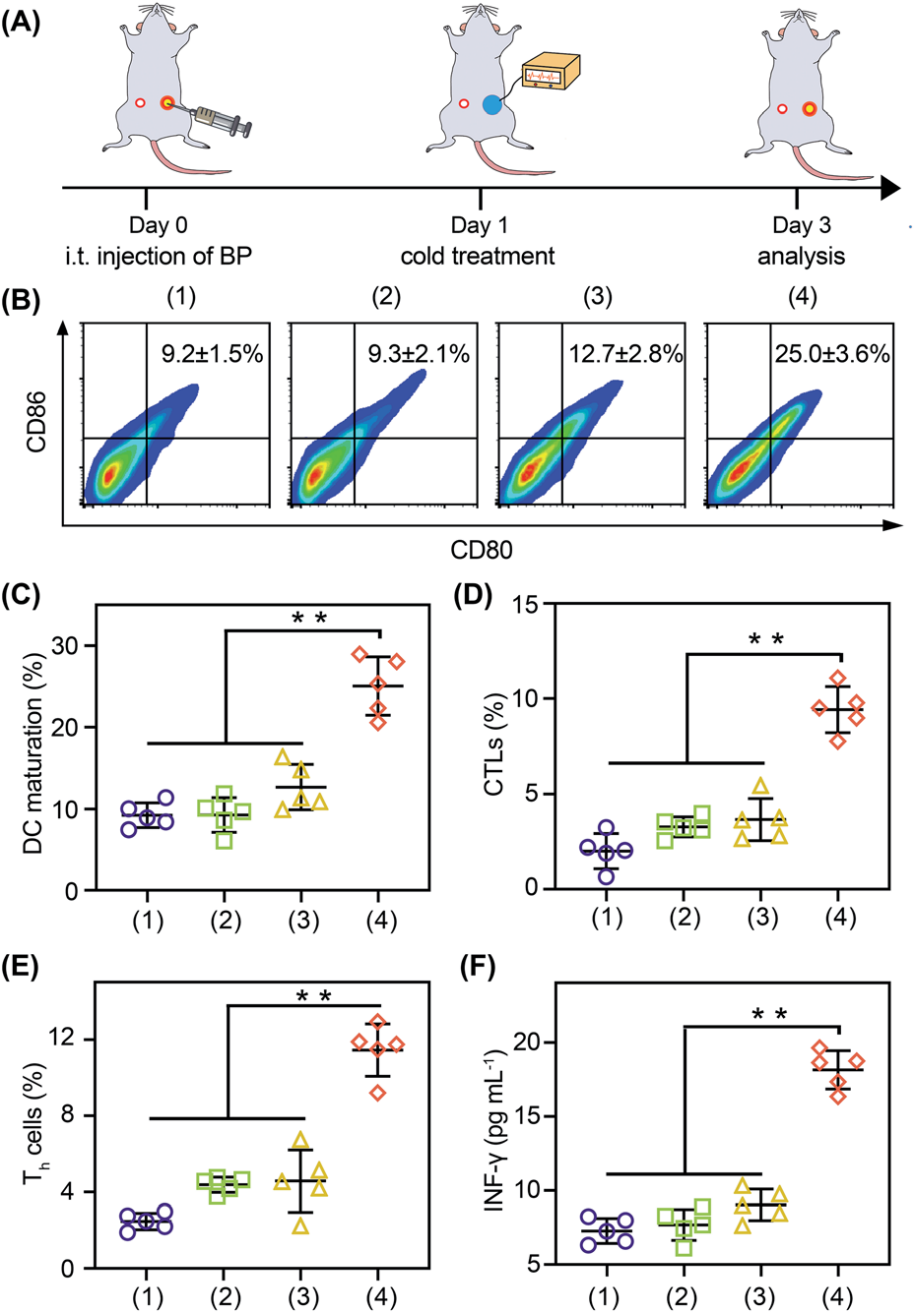
Cold-catalytic antitumor immunity with BP nanosheets *in vivo*. (A) Schedule for the detection of *in vivo* immune activation. (B) Flow cytometry analysis of dendritic cell (DC) maturation. (C) Matured dendritic cells (CD80^high^CD86^high^) were calculated according to flow cytometry. (D) and (E) Intratumoral activation of cytotoxic T lymphocytes (CTLs) and T helper (T_h_) cells in distant tumors. (F) Serum INF-γ was detected after different treatments. Groups: (1) PBS, (2) Δ*T*, (3) BP nanosheets, and (4) BP nanosheets + Δ*T*. The data were presented as mean ± SD (*n* = 5, **p < 0.01).

With the thermoelectric cooler as the cold source, we studied the *in vivo* immune modulating activity of BP nanosheets (Fig. 6A). After the cold-catalytic therapy of BP nanosheets, the percentage of M1 macrophages was significantly increased by 112.2%, and the secretion of proinflammatory cytokines (*i.e.*, TNF-α and IL-6) was up-regulated by 89.9% and 54.2% (Fig. S23 and S24†). Meanwhile, the percentage of M2 macrophages and the level of the anti-inflammatory cytokine IL-10 were decreased by 34.2% and 28.9% (Fig. S23†), respectively. In order to further explore the antitumor immune response, dendritic cell maturation and intratumoral T cell infiltration were analyzed by flow cytometry (Fig. 6B–E). Under the cold-catalytic treatment of BP nanosheets, the maturation rate of dendritic cells was elevated 2.7-fold, which could be attributed to the high ratio of immune cell death (Fig. 6C). Afterwards, T cells were activated and infiltrated into tumors to mediate adoptive immunity. Since the pro-inflammatory cytokines (*e.g.*, TNF-α and IL-6) could keep the activation of T cells within tumors,^38,39^ the infiltration of T cells was significantly increased by 6.5 and 4.2 times in primary tumors and distant tumors, respectively (Fig. S25†). Especially in the distant tumors, the populations of T helper cells and cytotoxic T lymphocytes were individually increased by about 4.6- and 4.7-fold (Fig. 6D, E and S25, S26). As a subpopulation of T cells, regulatory T cells (Tregs) mediated immunosuppression.^40^ After the cold-catalytic therapy, the relative population of Tregs in the CD4^+^ T cells was decreased by 89.8% and 44.6% in both primary and distant tumors (Fig. S27†). Furthermore, the serum level of INF-γ was also elevated by 149.4%, indicative of the successful activation of the antitumor immunity (Fig. 6F). All the results demonstrate the immunomodulating activity of BP nanosheets through cold-catalytic elevation of intratumoral oxidative stress.

**Fig. 7:**
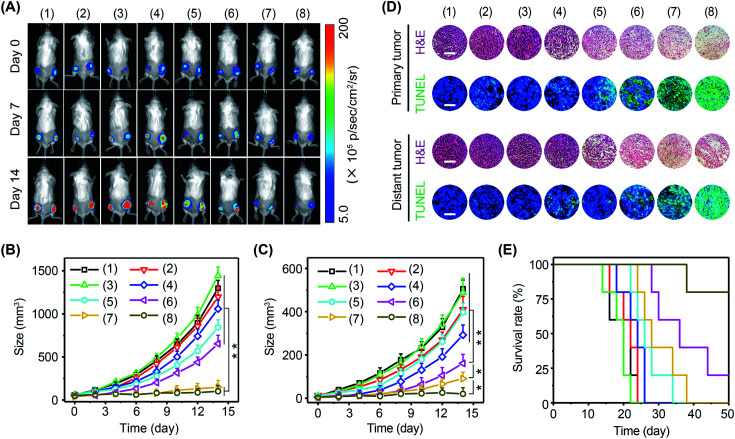
Cold-catalytic antitumor immunotherapy in tumor-bearing mice. (A) Representative bioluminescence images of 4T1-Luc tumor bearing mice in different groups: (1) PBS, (2) αPD-1, (3) Δ*T*, (4) Δ*T* + αPD-1, (5) BP nanosheets, (6) BP nanosheets + αPD-1, (7) BP nanosheets + Δ*T*, and (8) BP nanosheets + Δ*T* + αPD-1. (B) Growth curves of primary tumors and (C) distant tumors under different treatments. (D) Primary tumors and distant tumors were histologically examined through H&E and TUNEL staining (scale bars = 200 μm). (E) Survival rates of mice within 50 days post different treatments.

Immune cells are able to distinguish normal cells from foreign cells through immune checkpoint proteins to avoid self-attack. However, tumor cells can take advantage of immune checkpoint proteins to avoid immune recognition. In this regard, immune checkpoint inhibitors, such as PD-1 antibody (αPD-1), have been approved by the Food and Drug Administration (FDA) to boost immune recognition of tumors. Unfortunately, the immune suppression of tumors seriously impairs the performance of immune checkpoint inhibitors. Encouraged by the excellent immunomodulating performance of BP nanosheets, it was expected that the combination of αPD-1 and BP nanosheets could effectively activate antitumor immunity to arrest tumor growth and metastasis. The experiments were performed in 4T1-Luc tumor-bearing mice with eight groups: (1) blank control, (2) αPD-1, (3) Δ*T*, (4) αPD-1 + Δ*T*, (5) BP nanosheets, (6) BP nanosheets + αPD-1, (7) BP nanosheets + Δ*T*, and (8) BP nanosheets + αPD-1 + Δ*T*. Tumor growth was monitored *via* a bioluminescence imaging system every other day (Fig. 7A–C and S28, S29†). For mice without treatment, due to the absence of effector T cells in a tumor immunosuppressive environment, both primary tumors and distant tumors grew rapidly regardless of the addition of αPD-1. For mice in group (8), the growth of primary tumors was efficiently arrested after the cold-catalytic immunotherapy of BP nanosheets, probably owing to the pyroelectrically catalyzed oxidative stress in tumors (Fig. 5G). Furthermore, because of the activation of antitumor immunity by BP nanosheets under cold conditions, distant tumors were also remarkably eradicated with a barely detectable bioluminescence signal on day 14 (Fig. 7C and S28†). Histological analysis (Fig. 7D) revealed a large number of dead tumor cells with nuclear condensation and DNA fragmentation after the cold-catalytic immunotherapy of BP nanosheets. In addition, the survival rate of tumor-bearing mice was obviously elevated from <20% to 80% by cold-catalytic immunotherapy (Fig. 7E).

**Fig. 8:**
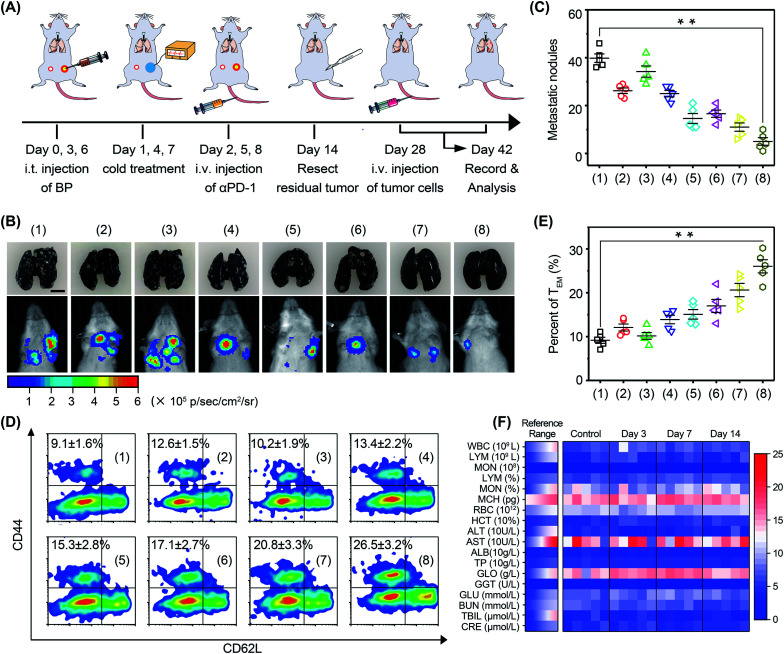
Immune memory effect against tumor metastasis. (A) Schedule for *in vivo* studies. (B) Representative bioluminescence images and photographs of metastatic nodules in the lungs of mice rechallenged with 4T1-Luc tumor cells (scale bar = 5 mm). All groups: (1) PBS, (2) αPD-1, (3) Δ*T*, (4) Δ*T* + αPD-1, (5) BP nanosheets, (6) BP nanosheets + αPD-1, (7) BP nanosheets + Δ*T*, and (8) BP nanosheets + Δ*T* + αPD-1. (C) Statistical results of tumor nodules in the lungs. (D) Flow-cytometry analysis of effector memory T cells (CD62L^low^CD44^+^) in splenic lymphocytes. (E) Percentages of T_EM_ in the splenic lymphocytes of mice. (F) Blood biochemistry analysis of the mice after intravenous injection of BP nanosheets, including white blood cells (WBC), lymphocytes (LYM), monocytes (MON), mean corpuscular hemoglobin (MCH), red blood cells (RBC), hematocrit (HCT), alanine aminotransferase (ALT), aspartate transaminase (AST), albumin (ALB), total protein (TP), globulin (GLO), gamma-glutamyl transpeptidase (GGT), glucose (GLU), blood urea nitrogen (BUN), total bilirubin (TBIL), and creatinine (CRE). *p < 0.05, **p < 0.01.

In order to investigate the anti-metastasis and anti-recurrence performance, mice were rechallenged with 4T1-Luc tumor cells on day 28 (Fig. 8A). Since 4T1-Luc tumors are readily metastasized to the lungs, pulmonary tumor nodes in mice were monitored *via* bioluminescence imaging (Fig. 8B). The results showed that the luminescence signal in the mice was very faint after cold-catalytic immunotherapy, while the luminescence signal of the other groups was bright in the breast area. Mouse lungs were extracted and stained with Indian ink to count pulmonary tumor nodes (Fig. 8B and C). The images clearly showed that BP nanosheet-based cold-catalytic immunotherapy inhibited the formation of pulmonary tumor nodes. By contrast, a large number of lung tumor nodules can be clearly observed in the mice of the other groups. Moreover, according to the flow cytometry analysis, effector memory T (T_EM_) cells in mice were increased 2.9-fold after cold-catalytic immunotherapy, suggesting the successful fabrication of the immune memory effect (Fig. 8D and E). During the treatments, all mice maintained normal body weight (Fig. S29†). The hematoxylin and eosin (H&E) staining results revealed the minimal damage of major organs after the cold-catalytic immunotherapy (Fig. S30†). In addition, BP nanosheets themselves showed little cytotoxicity towards both tumor cells and normal cells (Fig. S31†). The hemolysis assay of BP nanosheets was performed, and the results proved that the BP nanosheets induced <2% of hemolysis (Fig. S32†). Furthermore, blood cell populations and biochemical indices were within the normal range (Fig. 8F). For *in situ* vaccination, partial vaccination agents may diffuse into blood circulation and accumulate in the body, giving rise to a potential safety risk. We found that most BP nanosheets could be metabolized and cleared from the body even after intravenous administration (Fig. S33†). Therefore, BP nanosheet-based cold-catalytic immunotherapy was demonstrated with high biosafety for future clinical applications. Besides, since BP nanosheets are susceptible to degradation in the presence of O_2_, we studied the stability of BP nanosheets under mimicking physiological conditions. BP nanosheets were gradually degraded into non-toxic phosphate ions within one week (Fig. S34†). Notably, ∼80% of BP nanosheets preserve their intact structure under mimicking physiological conditions for more than 24 h (Fig. S34†). Since the cold-catalytic therapy was conducted 24 h after the administration of BP nanosheets, we believe that BP nanosheets could meet the stability requirement for *in situ* vaccination.

## Conclusions

In summary, with BP nanosheets as an example, we present a new strategy for tumor cold-catalytic immunotherapy. Due to the non-centrosymmetric and semiconducting nanostructure, BP nanosheets were demonstrated to have strong pyroelectricity. When subjected to cold treatment (4–37 °C), BP nanosheets catalyzed the generation of ROS to elevate intracellular oxidative stress, which induced serious mitochondrial damage and subsequent immunogenic cell death. During this process, tumor associated antigens were leaked from tumor cells for *in situ* vaccination. Meanwhile, the tumoral oxidative stress induced by BP nanosheets reprogramed the immunosuppressive tumor microenvironment. As a result, the host immune system was trained to successfully build systematic antitumor immunity to eradicate tumor metastasis. Moreover, the immune memory effect significantly inhibited tumor recurrence in mice, providing the host with long-term antitumor immunity. With a thermoelectric cooler as the cold source, an interventional device was constructed to precisely control the cold-catalytic immunotherapy. This work investigated a new kind of immunomodulating agent based on pyroelectric nanocatalysts, raising the prospects of cold-catalytic immunotherapy of cancer. Moreover, the high biosafety of cold, together with its high conductive depth (>10 mm) and spatio-temporal tunability, provides cold-catalytic immunomodulating nanoagents more opportunities as an *in situ* vaccination for practical applications. Our strategy can be straightforwardly extended to other cold-catalytic nanoagents, offering a general method for immunomodulation. We envision that cold-catalytic nanoagents would open up new opportunities to develop catalytic nanomedicine with vast biomedical applications.

## Data availability

All experimental and characterization data are available in the ESI.†

## Author contributions

X. Wu and M. Wen completed the conceptualization and investigation of this work. Y. Zou, X. Gao, and C. Wei contributed to the methodology and validation. R. Liu, J. Li, L. Wang and X. Li provided resources and visualization. Y. Liu contributed to supervision. W. Chen contributed to supervision, writing and editing the manuscript.

## Conflicts of interest

There are no conflicts to declare.

## Supplementary Material

SC-013-D2SC01894B-s001
