# Supplementary material for: Cold-catalytic antitumor immunity with pyroelectric black phosphorus nanosheets
Source: Chem Sci. 2022 May 17;13(23):6842–51. doi: 10.1039/d2sc01894b (PMC9200116; doi:10.1039/d2sc01894b)
Supplement: SC-013-D2SC01894B-s001 [file SC-013-D2SC01894B-s001.pdf]

Electronic Supplementary Material (ESI) for Chemical Science.
This journal is © The Royal Society of Chemistry 2022

Electronic Supporting Information

Cold-Catalytic Antitumor Immunity with Pyroelectric Black Phosphorus Nanosheets

Xianbo Wu†,[a] Mei Wen†,[a] Yuyan Zou,[a] Xinyu Gao,[a] Chuanwan Wei,[b] Renyu Liu,[c] Jianghua Li,[a] Long Wang,[c] Xilong Li,[d] You-Nian Liu,[a] and Wansong Chen*[a]

a. Hunan Provincial Key Laboratory of Micro & Nano Materials Interface Science, College of Chemistry and Chemical Engineering, Central South University, Changsha, Hunan 410083 (China) E-mail: chenws@csu.edu.cn.
b. School of Chemistry and Chemical Engineering, University of South China, Hengyang, Hunan 421001 (China).
c. Xiangya Hospital, Central South University, Changsha, Hunan 410083 (China).
d. Hefei National Laboratory for Physical Science at Microscale, University of Science and Technology of China, Hefei 230026 (China)
† These authors contributed equally to this work.

1. Experimental procedures

1.1 Materials

BP crystals and Cy5.5 were provided by Ruixi Biological Technology (Xi'an, China). Rhodamine B conjugated polyethylene glycol thiol was supplied by Yare Biotech (Shanghai, China). *N*-methyl-*2*-pyrrolidone (NMP), *2',7'*-dichlorodihydrofluorescein diacetate (DCFH-DA), terephthalic acid (TA) were purchased from Heowns Biochemical Technology (Tianjin, China). Coumarin-3-carboxylic acid (3-CCA) and D-luciferin potassium salt were bought from Yuanye Biotechnology (Shanghai, China). Lipopolysaccharides (LPS) from Escherichia coli O111:B4 were provided by Sigma (Darmstadt, Germany). Mouse interleukin 4 (IL-4) recombinant protein, calcein acetoxymethyl (calcein-AM), propidium iodide (PI), Annexin V-FITC/PI apoptosis detection kit, ATP assay kit, JC-1 staining kit, lactate dehydrogenase (LDH) assay kit, and D-luciferin were supplied by Beyotime Institute of Biotechnology (Haimen, China). Anti-calreticulin antibody (cat. ab92516), anti-HMGB-1 antibody (cat. ab79823), Alexa Fluor 647-conjugated secondary antibody (cat. ab150079), anti-NF-κB p65 (cat. ab207297), anti-phospho-NF-κB p65 (cat. ab239882), anti-IKKα/β (cat. ab178870), anti-phospho-IKKα/β (cat. ab194528), anti-histone H3 (cat. ab1791),

anti-GAPDH (cat. ab8245), and HRP-conjugated goat anti-rabbit secondary antibody (cat. ab6721) were purchased from Abcam (Cambridge, UK). PE-conjugated anti-mouse CD80 (cat. 104708), APC-conjugated antimouse CD86 (cat. 105011), FITC-conjugated anti-mouse CD206 (cat. 141704), APC-conjugated anti-mouse CD3 (cat. 100236), PE-conjugated anti-mouse CD4 (cat. 100408), FITC-conjugated anti-mouse CD8a (cat. 100706), Alexa Fluor 488 anti-mouse Foxp3 (cat. 126405), PerCP/Cy5.5 anti-mouse CD11c (cat. 117327), PerCP/Cy5.5 anti-mouse F4/80 (cat. 123128), PE-conjugated anti-mouse CD44 (cat. 103007), and PerCP/Cy5.5 anti-mouse CD62L (cat. 104431) were obtained from Biolegend (San Diego, USA). Anti-mouse PD-1 (cat. BE0146-5) was supplied by Bioxcell (West Lebanon, USA). Nuclear and cytoplasmic protein extraction kit, Membrane Protein Extraction Kit, Bradford protein assay kit and BeyoECL Plus kit were bought from Sangon Biotech (Shanghai, China). TNF-α ELISA kit, IL-6 ELISA kit, IL-10 ELISA kit, and IFN-γ ELISA kit were purchased from Neobioscience Technology (Shenzhen, China). L-012 was bought from Wako Chemicals (Richmond, USA). The phosphate assay kit (cat. 21665) was purchased from Biolite (Xi'an, China).

1.2 Synthesis of BP nanosheets

BP nanosheets were synthesized *via* a liquid exfoliation technique. Briefly, NaOH (0.5 g) was added into 50 mL of NMP and stirred at room temperature for 20 min. The supernatant was collected and mixed with BP crystals (10 mg) for ultrasonic exfoliation in an ice water bath. After 6 h of exfoliation, the mixture solution was centrifuged (1000 *g*, 5 min) to collect the supernatant. The obtained BP nanosheets in the supernatant were pelleted (12,000 *g*, 10 min) and washed with water twice to remove residual NMP.

1.3 Characterization of BP nanosheets

The morphology of the obtained BP nanosheets was observed with a transmission electron microscopy (JEOL, JEM-2100F, 200 kV). The thickness of BP nanosheets was measured by an atomic force microscope (Veeco, NanoMan). Hydrodynamic diameter (DLS), polydispersity index (PDI) and zeta potential were characterized by a Zetasizer Nano-ZS instrument (ZEN3600 Malvern, UK). The XPS spectra were detected on an X-ray photoelectron spectrometer (ThermoFischer, ESCALAB 250XI, USA) with an Al Kα ray (hv = 1486.6 eV) as the excitation source. The X-ray diffraction (XRD) patterns were measured through a RigaKu X-ray diffractometer (SIMENS D500, Switzerland). Raman scattering spectrum was tested through a Raman microspectrometer (Jobin

Yvon LabRam-010, France). Fluorescence spectra were conducted on a fluorescence spectrometer (Hitachi F-4600, Japan). Piezoresponse force microscopy (PFM) data were recorded with a NT-MDT NTEGRA atomic force microscope (NT-MDT, Russia). The UV-vis absorption spectra were recorded by a UV−2450 spectrometer (Shimadzu, Japan). The UV-vis diffuse reflectance spectra were obtained using a UV-2600 spectrophotometer (Shimadzu, Japan).

1.4 Pyro-electrochemical measurements

BP nanosheets (0.5 mg) were dispersed in 10 μL of ethanol and then mixed with 10 μL of 5% Nafion perfluorinated resin solution. The mixture solution was dropped on a glassy carbon electrode. After drying, the electrode was immersed in 0.5 M Na_2SO_4 solution. The pyroelectric current and voltage were recorded on an electrochemical workstation (Kerui RST5200, China). The measurement was performed with Ag/AgCl as a reference electrode and Pt wire as a counter electrode. The cold temperature was controlled by a thermoelectric cooler (TES1-04903, HongDaEr, China), and powered by a direct current power supply (VC3003A, VICTOR, China). The temperature was decreased from 37 ^{o}C to 4 ^{o}C for 5 min and then transferred to a 37 ^{o}C water bath for another 5 min to recover temperature. Both the pyroelectric currents and pyroelectric potentials were recorded during 4 cycles of temperature variations. The pyroelectric current was measured at 0.02 V vs Ag/AgCl, and pyroelectric potential was measured at 5 mA.

1.5 ROS detection

The radicals in cold-catalysis of BP nanosheets were measured through an electron spin resonance (ESR) spectrometer (Bruker model A300, Bruker Biospin GmbH, Germany). Briefly, BP nanosheets were treated with temperature variations between 4 ^{o}C and 37 ^{o}C for 20 cycles in water or DMSO. ESR spectra of the mixture solution were collected with DMPO as a spin trap agent.

•OH was quantitatively detected with terephthalic acid (TA) as a fluorescent probe. TA (0.83 mg) was dissolved in 10 mL of NaOH aqueous solution (2 mM), and then 100 μL of TA solution was mixed with 1 mL of BP nanosheets (50 μg mL^{-1}) in water. The solution was treated with a thermoelectric cooler (4 ^{o}C) for 5 min. Afterwards, the samples were transferred into a 37 ^{o}C water bath and incubated for 5 min to recover temperature. The temperature was varied for different cycles (0, 5, 10, 15, 20 cycles), and the fluorescence spectra were measured on a fluorescence spectrometer.

To quantify $\bullet O_2^-$ production during the pyro-catalytic process, 100 µL of NBT solution (0.01 mM) in DMSO was mixed with 1 mL of BP nanosheets (50 µg mL^{-1}) in water. Similar as above mentioned procedure, the temperature was varied between 37 °C and 4 °C for different cycles (0, 5, 10, 15, 20 cycles), and the absorption at 680 nm was measured on a UV−2450 spectrometer.

DCFH-DA was utilized to detect the total production of ROS in the solution. Typically, 0.5 µL of DCFH-DA (10 mM) solution was mixed with 20 µL of NaOH solution (10 mM) for hydrolysis into DCFH. Afterwards, the mixture was transferred to 10 mL of BP nanosheets (50 µg mL^{-1}) in PBS (10 mM, pH 7.4). Both a thermoelectric cooler (0.6 W ~ 7.5 W) and a 37 °C water bath were used for temperature variation between 4 °C and 37 °C. The fluorescence intensity of the mixture was recorded on a fluorescence spectrometer.

1.6 Energy band analysis

The mixed solution of BP nanosheets was dropped on the glassy carbon electrode according to method 1.4. After drying, the electrode was immersed in 0.5 M Na_2SO_4 solution. The Mott-Schottky curve was measured by an electrochemical workstation (Kerui RST5200, China) with Ag/AgCl reference electrode and Pt wire counter electrode placed in an electrolytic cell. The Tauc plot was calculated on UV-vis diffuse reflectance spectrum data as following equation.[1-2]

$$[F(R_\infty)]^n = A(h\nu - E_g) \qquad (5)$$

$h\nu$, A and E_g represent the values of photon energy, proportionality constant and band gap respectively. The direct transition of n = 2 or the indirect transition of n = 1/2 can be defined by the Kubelka-Munk function $F(R_\infty)$[1-2]:

$$F(R_\infty) = \alpha/S = (1 - R)^2/2R \qquad (6)$$

where α, S and R are absorption, scattering coefficient, and diffuse reflection, respectively.

The XPS valence band spectrum were recorded on an X-ray photoelectron spectrometer (ThermoFischer, ESCALAB 250XI, USA) with an Al Kα ray ($h\nu$ = 1486.6 eV) as the excitation source.

1.7 Cell lines and animals

4T1 breast cancer cells, L929 fibroblast cell, A549 lung carcinoma cells, and J774A.1 macrophages were purchased from Xiangya Hospital, Central South University (Changsha, China). Fetal bovine serum (FBS), Dulbecco's modified Eagle medium (DMEM), trypsin, penicillin streptomycin and PBS were obtained from Gibco Invitrogen (Beijing, China). Male BALB/c mice (5

weeks old) were purchased by Hunan SJA Laboratory Animal Co. Ltd. (Changsha, China). All animal experiments were conducted in accordance with the regulations approved by the Laboratory Animal Center of Xiangya School of Medicine, Central South University.

1.8 Cellular uptake study

To prepare rhodamine B-labeled BP (RhB-labeled BP), Rhodamine B conjugated polyethylene glycol thiol (1 mg mL^{-1}) was mixed with BP nanosheets (100 μg mL^{-1}) in water. Then, the RhB-labeled BP nanosheets were centrifuged (12000 *g*, 5 min) and washed four times. 4T1 cells and J774A.1 macrophages were incubated with RhB-labeled BP (50 μg mL^{-1}) for different periods (0 h, 1 h, 2 h, 3 h, and 4 h). Afterwards, 4T1 cells and J774A.1 macropahges were washed with PBS twice, and the nuclei were stained with DAPI at 37 ^{o}C for 30 min. Cells were imaged under an inverted fluorescence microscope (Olympus IX-83, Japan). and the fluorescence intensity was analyzed by a flow cytometer (NovoCyte 2040, Agilent, USA).

1.9 Intracellular oxidative stress and oxidative damage

4T1 cells were incubated with BP nanosheets (50 μg mL^{-1}) for 12 h, and then subjected to temperature variations between 4 ^{o}C and 37 ^{o}C for 20 cycles as mentioned above. Intracellular oxidative stress was then measured with DCFH-DA (50 μg mL^{-1}) as a probe. In order to simulate a hypoxic environment, 4T1 cells were cultured in a medium bubbled with N_2, and sealed with paraffin oil. The other operations were the same as above. To detect mitochondrial integrity, cells were stained with JC-1 (10 μg mL^{-1}) and Hoechst 33342 (10 μg mL^{-1}) for 30 min. Cells were imaged under an inverted fluorescence microscope (Olympus IX-83, Japan), and the fluorescence intensity was analyzed by CellSens software (Olympus, Japan).

1.10 Immunogenic cell death

To detect immunogenic cell death, 4T1 cells were incubated with BP nanosheets (50 μg mL^{-1}) and then subjected to temperature variations between 4 ^{o}C and 37 ^{o}C for 20 cycles. After incubated for another 12h, ATP level in the supernatant of each well was measured by the ATP assay kit following the manufacturer's protocol. For calreticulin immunostaining, cells were stained with anti-calreticulin at 4 °C for 12 hours, and incubated with AF647-conjugated secondary antibody at 37 °C for another 2 h. For HMGB-1 immunostaining, cells were stained with anti-HMGB-1 antibody at 4 °C for 12 hours, and incubated with AF647-conjugated secondary

antibody at 37 °C for 2 h. After nucleus staining with DAPI at 37 °C for 30 min, cells were imaged under a confocal laser scanning microscope (ZEISS LSM880, Germany).

4T1 cells were incubated with BP nanosheets (50 μg mL^{-1}) and then subjected to temperature variations between 4 °C and 37 °C for 20 cycles. After incubation for 12h, cells were collected for cytoplasmic/membrane protein extraction with an extraction kit. Sodium dodecyl sulfate polyacrylamide gel electrophoresis (SDS-PAGE) was conducted with 10% arylamide gels for protein separation. The gels were then transferred to polyvinylidene fluoride (PVDF) membranes (Millipore, USA) at a current of 290 mA for 2 h. To prevent non-specific binding of proteins to antibodies, PVDF membranes were blocked with skimmed milk (5 wt%) for 3 h. Afterwards, the PVDF membranes were stained with the primary antibodies at 4 °C overnight and with the secondary antibodies at room temperature for 2 h. Protein bands were detected with a BeyoECL Plus kit and imaged with a chemiluminescence gel imaging system (Universal Hood II, BIO-RAD, USA).

1.11 Live and dead staining

4T1 cells were incubated with BP nanosheets (50 μg mL^{-1}) for 12 h and then subjected to temperature variations between 4 °C and 37 °C for 20 cyles. After incubated for another 12h, all the cells were washed with PBS and stained with calcein-AM/PI at 37 °C for 20 min. Then, cells were washed with PBS twice and imaged under an inverted fluorescence microscope.

1.12 Cell apoptosis and necrosis assay

4T1 cells were incubated with BP nanosheets (50 μg·ml^{-1}) overnight, and subjected to temperature variations between 4 °C and 37 °C for 20 cycles, then cells were incubated for 12 h. To detect cell apoptosis, cells were collected and stained with Annexin V-FITC/PI for 20 min according to the manufacturer's protocol. The percentage of apoptotic cells was analyzed with a flow cytometer (NovoCyte 2040, Agilent, USA).

1.13 In vitro macrophage polarization

J774A.1 macrophages were polarized with LPS (1 μg mL^{-1}) or IL-4 (25 ng mL^{-1}) overnight to induce M1 or M2 polarization. M2 macrophages were treated with BP nanosheets (50 μg mL^{-1}), and subjected to temperature variations between 4 °C and 37 °C for 20 cycles, and then incubated for 4 h. After immunostaining with APC-labeled CD86 and FITC-labeled CD206 antibodies at 4 °C overnight, cells were detected with a flow cytometer for polarization analysis.

IL-6, TNF-α and IL-10 in the supernatants were collected for ELISA measurements following the manufacturer's protocols.

Since tumor cells were subjected to cold-catalytic oxidative stress prior to macrophages. In other words, tumor cells were exposed to cold-catalytic oxidative stress for a longer time than macrophages. To mimic this process *in vitro*, tumor cells were subjected to cold-therapy of BP nanosheets for 12 h; whereas macrophages were subjected to cold-therapy of BP nanosheets for 4 h. Afterwards, BP nanosheets were removed and macrophages were further incubated for another 8 h. The cell viability of macrophages was determined with LDH assay kit.

1.14 Immunoblotting analysis

Macrophages were treated with BP nanosheets (50 μg mL^{-1}) for 4 h, and then subjected to temperature variations between 4 ^{o}C and 37 ^{o}C for 20 cycles. Afterwards, macrophages were incubated for 4 h and collected for nuclear/cytoplasmic protein extraction with an extraction kit. Sodium dodecyl sulfate polyacrylamide gel electrophoresis (SDS-PAGE) was conducted with 10% arylamide gels for protein separation. The gels were then transferred to polyvinylidene fluoride (PVDF) membranes (Millipore, USA) at a current of 290 mA for 2 h. To prevent non-specific binding of proteins to antibodies, PVDF membranes were blocked with skimmed milk (5 wt%) for 3 h. Afterwards, the PVDF membranes were stained with the primary antibodies at 4 °C overnight and with the secondary antibodies at room temperature for 2 h. Protein bands were detected with a BeyoECL Plus kit and imaged with a chemiluminescence gel imaging system (Universal Hood II, BIO-RAD, USA).

1.15 Cold conduction

The experiment was carried out in accordance with the regulations approved by Medical Ethics Committee of Xiangya Hospital, Central South University. Written informed consent was obtained from the volunteer and documented prior to the experiments. The thermoelectric cooler was fixed on a volunteer hand and the cooler temperature was set at 4 ^{o}C. After exposure to the thermoelectric cooler for 5 min, human hand was imaged under a thermal imaging camera to record the temperature variation (FLIR C2, Teledyne FLIR, USA). A pork skin (10 mm thickness) was exposed to the thermoelectric cooler for 5 min, and temperature on the other side of the skin was monitored under the thermal imaging camera.

A 4 °C cold source was applied outside the skin, and the cold distribution within skin tissue

was simulated by COMSOL Multiphysics. The geometric model was built as a cuboid structure with length, width and height of 60mm, 60mm and 10mm, respectively. The structure parameters were set as following: density 1100 kg m^{-3}, thermal conductivity 0.9 W m^{-1} K^{-1}, specific heat capacity 3300 J Kg^{-1} K^{-1}.[3]

1.16 Cold-induced injury

To investigate the cold-induced injury, mice (n = 4) were treated with the thermoelectric cooler (4 °C, 5 min) for 20 cycles. Mice without cold treatment were taken as control. Following mouse euthanasia, the skin was collected and histologically examined through H&E staining.

1.17 In vivo ROS generation

4T1 tumor cells were subcutaneously injected into BALB/c mice to construct *in vivo* tumor model. When tumor size reached ~50 mm^3, mice were received the following different treatments (n = 5 in each group): (1) blank control, (2) ΔT, (3) BP, (4) BP + ΔT. BP nanosheets were intratumorally injected into mice at a dosage of 5 mg kg^{-1}. At 24 h post injection, mice in the group (2) and (4) were treated with the thermoelectric cooler (4 °C, 5 min) for 20 cycles at tumor sites. Then mice were received an intravenous injection of L-012 (75 mg kg^{-1}), and imaged on an *in vivo* imaging system (IVIS Lumina III, PerkinElmer, USA). Moreover, the thermal image of mouse with different cold time was measured by a thermal imaging camera (FLIR C2, Teledyne FLIR, USA).

1.18 In vivo macrophage polarization

Tumor-bearing mice were received various treatments as mentioned above. On day 3 post-injection, the tumor tissue was collected and homogenized to obtain a single-cell suspension. After immunostaining with PerCP/Cy5.5-labeled F4/80, APC-labeled CD86 and FITC-labeled CD206 antibodies, macrophage polarization was analyzed with a flow cytometer. Serum cytokines, including TNF-α, IL-6 and IL-10, were detected with corresponding ELISA kits.

1.19 Dendritic cell maturation and T cell infiltration within tumors

To construct a bilateral tumor-bearing mice model, 4T1 cells were firstly subcutaneously injected into the right flank and the left flank of mice to build primary and distant tumor models, respectively. When the size of primary tumors reached ~50 mm^3, mice were received the following different treatments (n = 5 in each group): (1) blank control, (2) ΔT, (3) BP, (4) BP + ΔT. Mice were received intratumoral injection of BP nanosheets (5 mg kg^{-1}). At 24 h post injection,

mice in group (2) and (4) were treated with the thermoelectric cooler treatment (4 °C, 5 min) for 20 cycles on the primary tumors. On day 3 post injection, tumors, spleens and blood were collected following mouse euthanization. After homogenization and hemolysis of erythrocytes, cell suspensions of tumors and spleens were obtained for immunostaining. Tumor cell suspensions were stained with APC-labeled CD3, PE-labeled CD4 and FITC-labeled CD8a antibodies. The splenic cell suspensions were stained with PerCP/Cy5.5-labeld CD11c, PE-labeled CD80 and FITC-labeled CD86 antibodies. All the cells were analyzed on a flow cytometer. Mice blood was centrifuged (1000 *g*, 5 min), and the serum IFN-γ level was measured with an ELISA kit.

1.20 In vivo antitumor effect

The experiment was conducted in bilateral tumor-bearing mice with eight groups (n = 5 in each group): (1) PBS, (2) αPD-1, (3) Δ*T*, (4) Δ*T* + αPD-1, (5) BP, (6) BP + αPD-1, (7) BP + Δ*T*, (8) BP + Δ*T* + αPD-1. When the size of the primary tumor reached ~50 mm^3, BP nanosheets were intratumorally injected into mice (5 mg kg^{-1}). On day 1, 4 and 7 post injection, the primary tumors in group (3) (4) (7) and (8) were treated with a thermoelectric cooler (4 °C, 5 min) for 20 cycles. On day 2, 5 and 8, mice in the group (4), (6) and (8) were received intravenous injection of αPD-1 (0.75 mg kg^{-1}). Tumor size was calculated with a formula: V = length × $width^2/2$, and monitored by an *in vivo* imaging system (LVIS Lumina III, PerkinElmer, USA) after intravenous injection of D-luciferin (150 mg kg^{-1}). Both tumor size and mouse body weight were recorded every other day. On day 14, all the mice were euthanized for tumor collection and histological examination, including TUNEL staining and hematoxylin & eosin (H&E) staining. To study mouse survival rate, tumor-bearing mice were treated as mentioned above. According to ethical requirement, mice were euthanized once their tumor size was over 2000 mm^3. All mice were euthanized on day 50 and the survival rate in each group was calculated.

1.21 Immune memory effect against tumor recurrence

4T1 tumor cells (1×10^7 cells) were subcutaneously injected into mice. The study was conducted with following groups (n = 10 in each group): (1) blank control, (2) αPD-1, (3) Δ*T*, (4) Δ*T* + αPD-1, (5) BP, (6) BP + αPD-1, (7) BP + Δ*T*, (8) BP + Δ*T* + αPD-1. When the size of primary tumor reached ~50 mm^3, mice were received an intratumoral injection of BP nanosheets (5 mg kg^{-1}) on day 0, 3 and 6. Mice in group (3), (4), (7) and (8) were treated with the thermoelectric cooler (4 °C, 5 min) for 20 cycles at 24 h post-injection. On day 2, 5 and 8 post injection, mice were

received intravenous injection of αPD-1 (0.75 mg·kg^{-1}). On day 14, all the tumors were surgically removed from mice. To demonstrate the generation of memory T cells, half of mice were sacrificed on day 28 to collect spleens. After homogenization and hemolysis of erythrocytes, the splenic cells were stained with APC-conjugated anti-mouse CD3, FITC-conjugated anti-mouse CD8a, PE-conjugated anti-mouse CD44, and PerCP/Cy5.5 anti-mouse CD62L. The population of memory T cells were analyzed with flow cytometry. Another half of mice were received intravenous injection of 4T1-Luc tumor cells (1× 10^6). On day 42, mice were received intravenous injection of D-luciferin and imaged by an *in vivo* imaging system. Afterwards, mice were euthanized and the lungs were stained with 15% India ink. The metastatic nodules in the lungs were imaged with a digital camera and counted for statistical analysis.

1.22 Stability and biosafety study

To determine the degradability of BP nanosheets, the nanosheets (50 μg·ml^{-1}) were dispersed in distilled water and DMEM cell culture medium, respectively. The solution was taken every 24 h to record UV-Vis absorption spectrum. Meanwhile, BP nanosheets were centrifuged at 12000 *g* for 10 min, and the concentration of phosphate ion in the supernatants was determined with the phosphate assay kit.

After *in vivo* cold-catalytic immunotherapy, all the mice were euthanized and the major organs were collected for histological hematoxylin & eosin (H&E) examination.

To study cytotoxicity of BP nanosheets, 4T1, L929 and A549 cells were incubated with BP nanosheets (50 μg·ml^{-1}) overnight. The supernatants of medium were collected for LDH detection with LDH assay kit. Cell viability was calculated with the following equation (7).

$$\text{Cell viability (\%)} = ([LDH]_{total}-[LDH]_{release})/[LDH]_{total} \times 100\% \qquad (7)$$

$[LDH]_{total}$ represents the total LDH within all the cells, and $[LDH]_{release}$ represents the LDH released into the supernatant.

Mouse erythrocytes were separated from the whole blood and washed with PBS three times. Erythrocytes (2 μL) were incubated with BP nanosheets (0, 25, 50, 100, 200 μg mL^{-1}) in PBS at 37 ^{o}C for 8 h. Then, the mixture solution was centrifuged at 1000 *g* for 5 min, and the supernatants were collected for absorption measured (λ = 540nm). The hemolysis rate was calculated with the following equation:

$$\text{Hemolysis (\%)} = (A / A_0) \times 100\% \qquad (8)$$

where, A is the absorption of supernatant after incubation with BP nanosheets, and A_0 is the absorption of hemolytic erythrocytes in pure water.

Mice were intravenously injected with BP nanosheets (5 mg kg^{-1}). On day 3, 7 and 14 post injection, mouse blood was collected and analyzed on an automated hematology analyzer (HF-3800, HLife, China). Meanwhile, blood biochemistry indexes were measured on a hematology chemistry analyzer (PointCare V2, MNChip, China).

To prepare Cy5.5-labeled BP nanosheets, 20 mL of Cy5.5 (0.2 mg mL^{-1}) aqueous solution was mixed with 20 mL of BP nanosheets (0.1 mg mL^{-1}) in water for 5 h. The excess Cy5.5 was removed through centrifugation (12,000 *g*, 10 min). To study the distribution and clearance of BP nanosheets, mice were received intravenous injection of Cy5.5-labeled BP nanosheets (5 mg kg^{-1}). The fluorescence of Cy5.5-labeled BP nanosheets in mice was monitored on an *in vivo* imaging system (IVIS Lumina III, PerkinElmer, USA).

1.23 Statistical analysis

Data were expressed as mean ±SD. Experiments were repeated three times in this work unless otherwise noted. A one-way analysis of variance was employed to analyzed the significance of the difference, and the statistical significance was defined as $^{*}p < 0.05$ and $^{**}p < 0.01$.

2. Supplementary figures

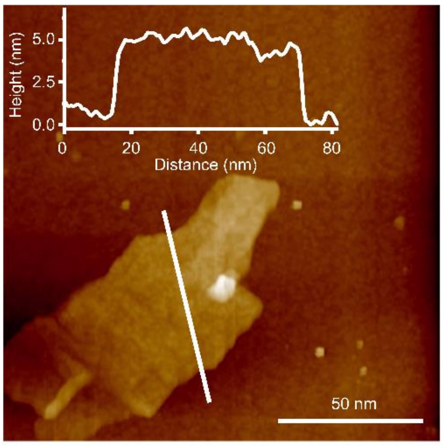

Figure S1. AFM image of BP nanosheets.

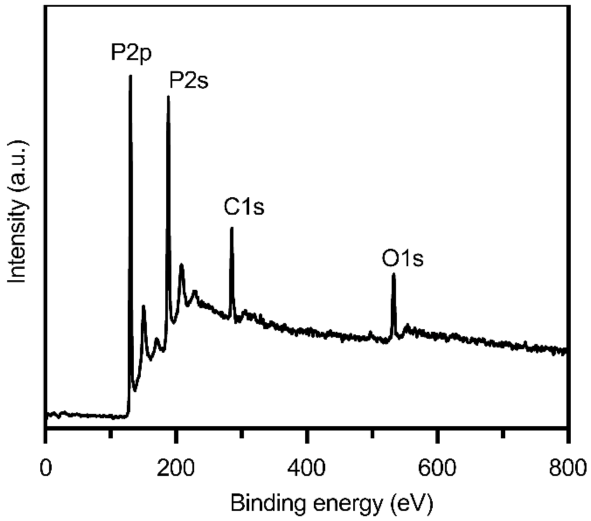

Figure S2. XPS spectra of BP nanosheets.

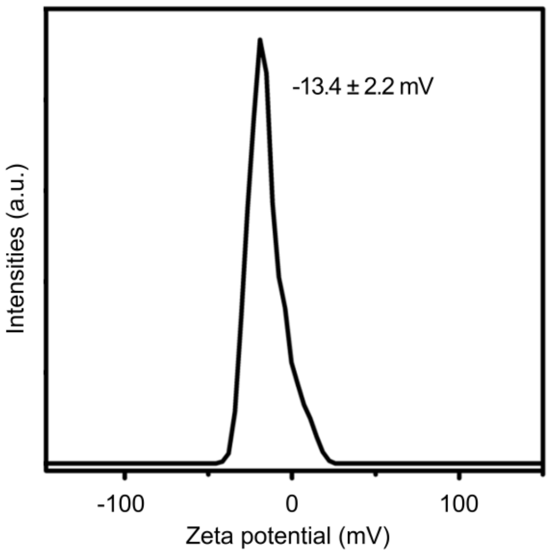

Figure S3. zeta potential of BP nanosheets.

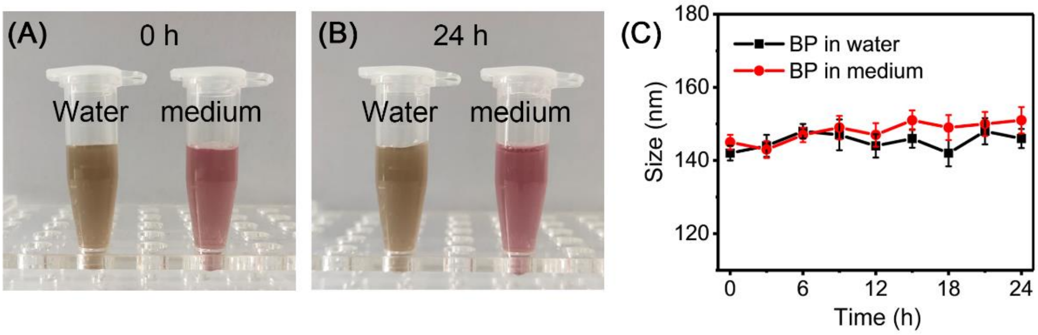

Figure S4. The photographs of BP nanosheets in water and DMEM cell culture medium at 0 h (A) and 24 h (B). (C) Dispersion stability of BP nanosheets in water and DMEM cell culture medium was monitored for 24 h.

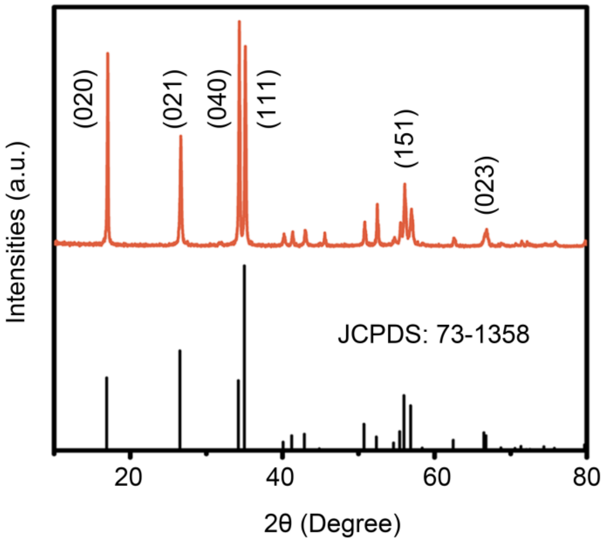

Figure S5. XRD pattern of BP nanosheets.

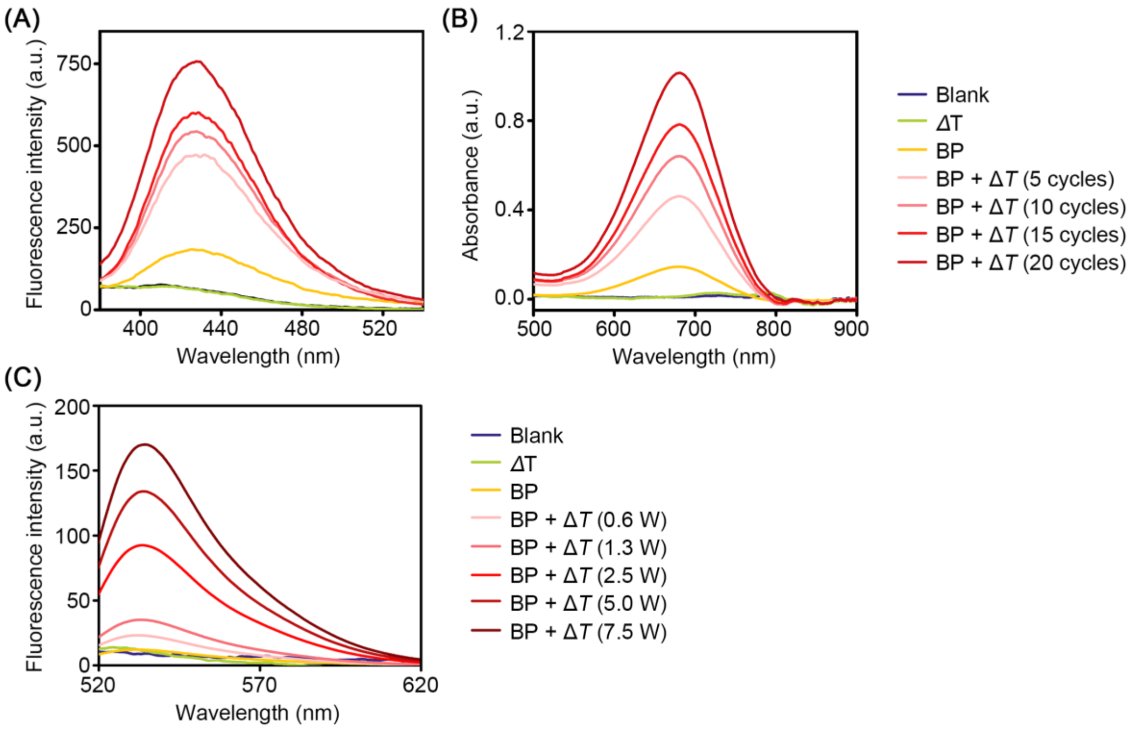

Figure S6. (A) Fluorescence spectra of TA for •OH detection at different cycling times (λ_{ex} = 315 nm). (B) UV-vis absorption spectra of NBT for $•O_2^-$ detection at different cycling times. (C) Fluorescence spectra of DCFH with a thermoelectric cooler as the cold source (λ_{ex} = 480 nm). The input power of the cooler was varied from 0.6 W to 7.5W.

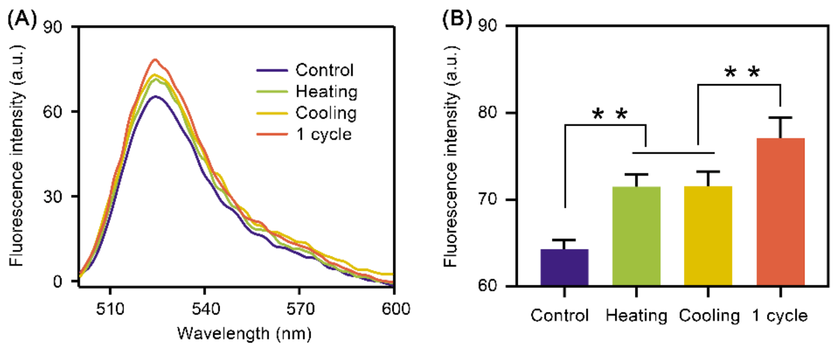

Figure S7. The fluorescence spectra of DCFH for ROS detection at separate cooling process, heating process and cycle process. ***p* < 0.01.

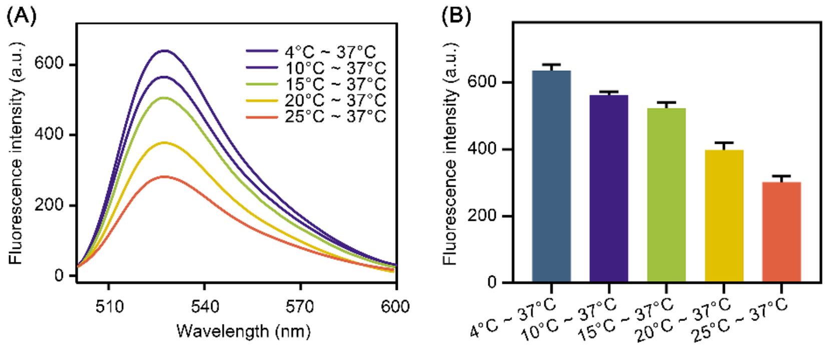

Figure S8. (A) Fluorescence spectra of DCFH for ROS detection after 20 cycles of temperature variations. (B) The maximum fluorescence intensity in different groups was compared based on the results of (A).

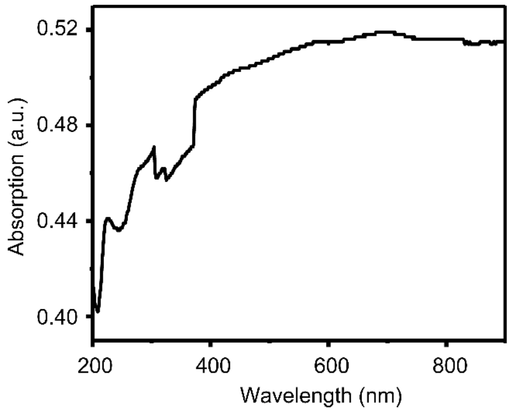

Figure S9. The UV-vis diffuse reflectance spectrum of BP nanosheets.

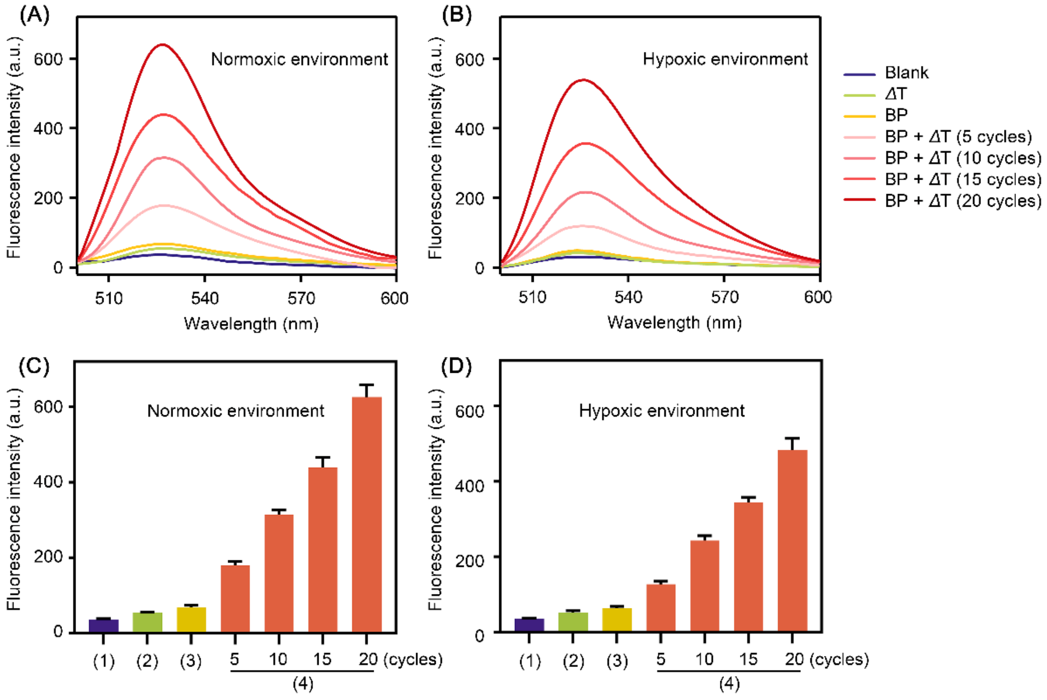

Figure S10. Fluorescence spectra of DCFH for ROS detection in normoxia environment (A) and hypoxia environment (B). (C−D) The maximum fluorescence intensity in different groups was compared based on the results of (A−B).

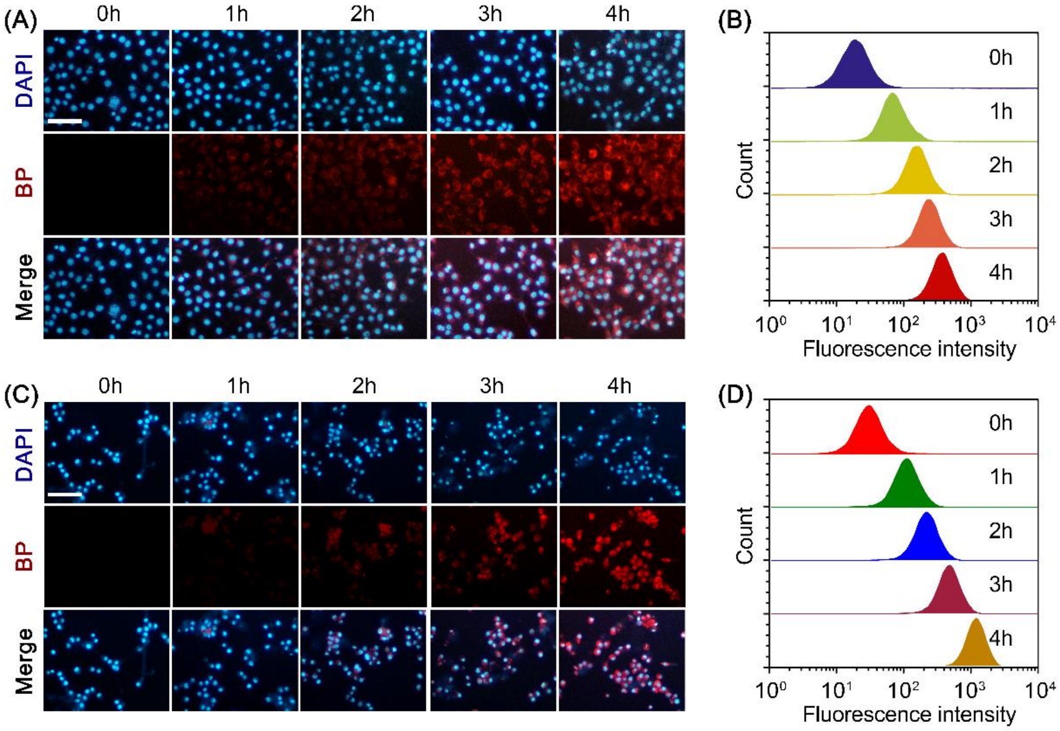

Figure S11. Cellular uptake of RhB-labeled BP nanosheets. (A) Fluorescence images of 4T1 tumor cells treated with RhB-labeled BP nanosheets at different time intervals (scale bars = 50 μm). (B) Flow cytometry analysis of fluorescence in 4T1 tumor cells. (C) Fluorescence images of J774A.1 macrophages treated with RhB-labeled BP nanosheets at different time intervals (scale bars = 50 μm). (D) Flow cytometry analysis of fluorescence in J774A.1 macrophages.

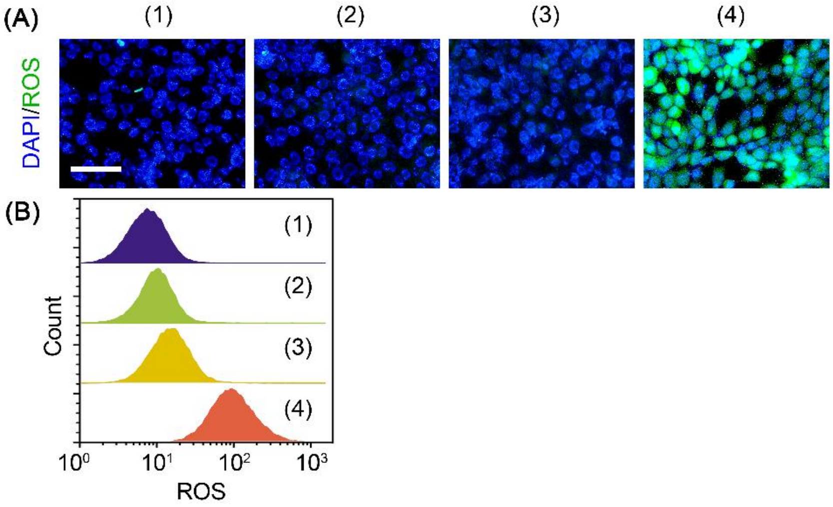

Figure S12. Fluorescence images of intracellular oxidative stress in hypoxia. Nuclei were stained by DAPI with blue fluorescence (scale bars = 50 μm). (B) Intracellular fluorescent intensity of oxidative stress was analyzed through flow cytometry. Groups: (1) PBS, (2) Δ*T*, (3) BP nanosheets, (4) BP nanosheets + Δ*T*.

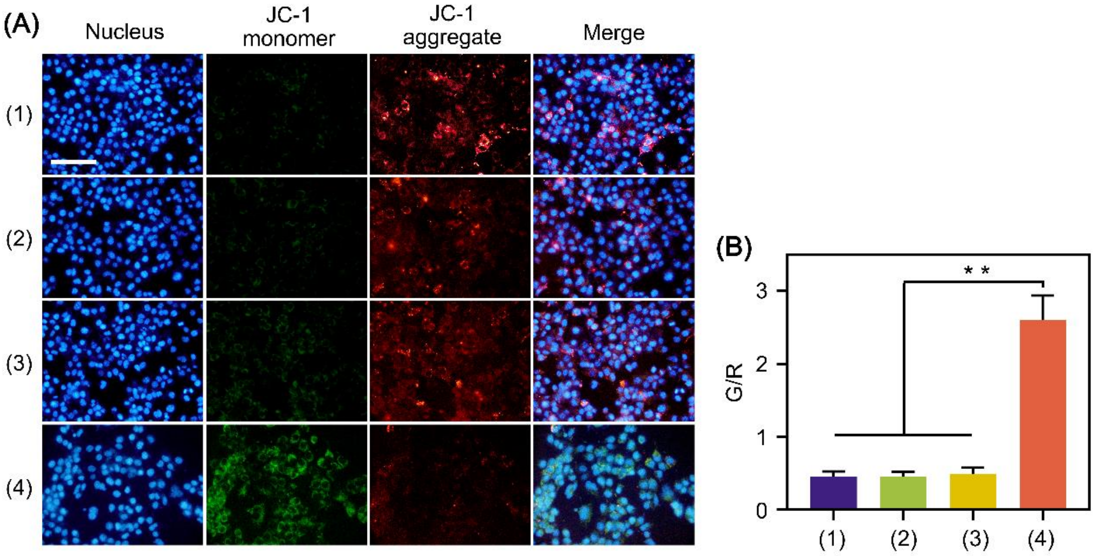

Figure S13. (**A**) JC-1 staining of 4T1 tumor cells after different treatments: (1) PBS, (2) Δ*T*, (3) BP

nanosheets, (4) BP nanosheets + Δ*T*. Scale bar = 100 µm. (B) The green to red fluorescence (G/R) ratio was measured using CellSens software (**$p < 0.01$).

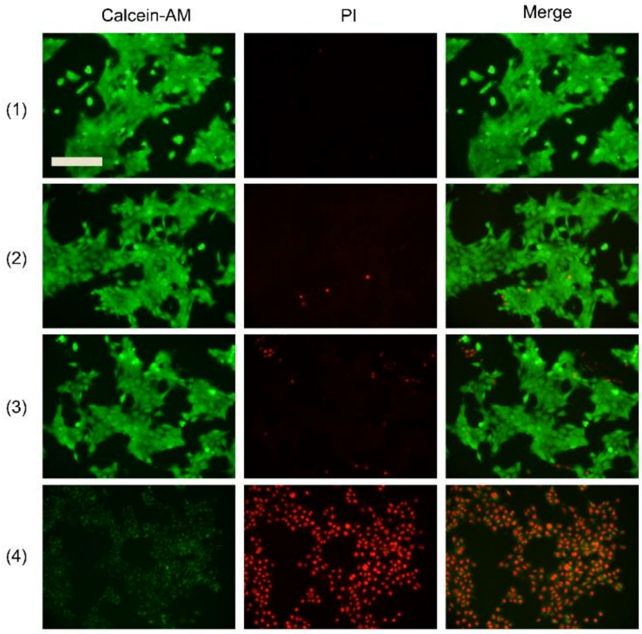

Figure S14. 4T1 cells were stained with calcein-AM (green) and PI (red) after different treatments: (1) PBS, (2) Δ*T*, (3) BP nanosheets, (4) BP nanosheets + Δ*T* (scale bar = 100 µm).

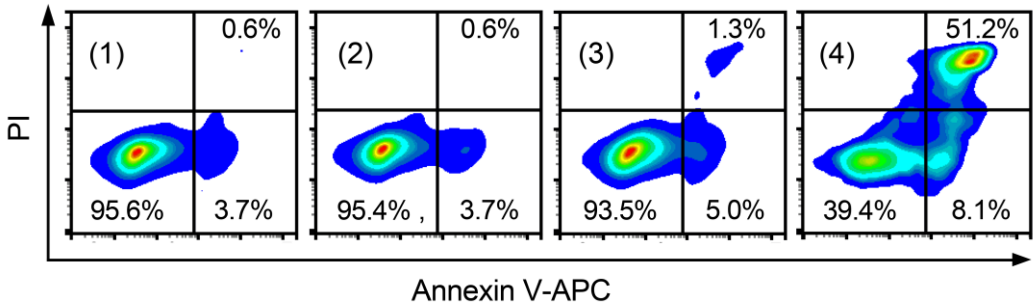

Figure S15. Flow cytometry analysis of apoptosis and necrosis of 4T1 cells through Annexin V/PI staining after different treatments: (1) PBS, (2) Δ*T*, (3) BP nanosheets, (4) Δ*T* + BP nanosheets.

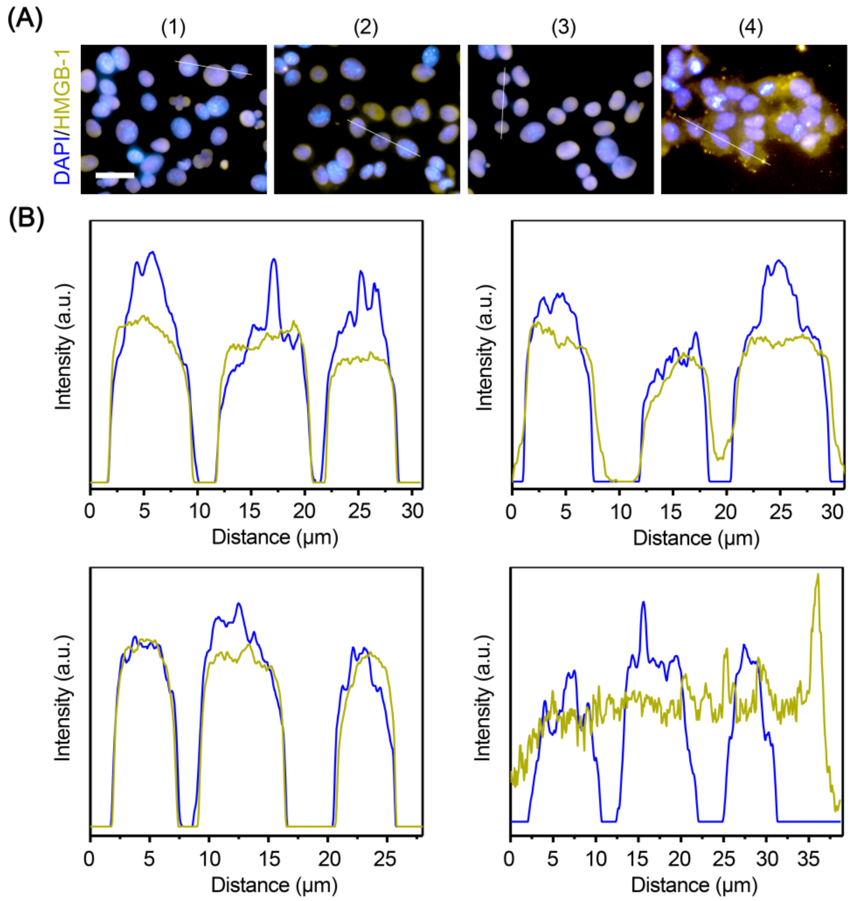

Figure S16. (A) Fluorescence images of HMGB-1. Nuclei were stained by DAPI with blue fluorescence after different treatments (scale bars = 20 μm). Groups: (1) PBS, (2) Δ*T*, (3) BP nanosheets, (4) BP nanosheets + Δ*T*. (B) The fluorescence distributions alone the white lines in (A).

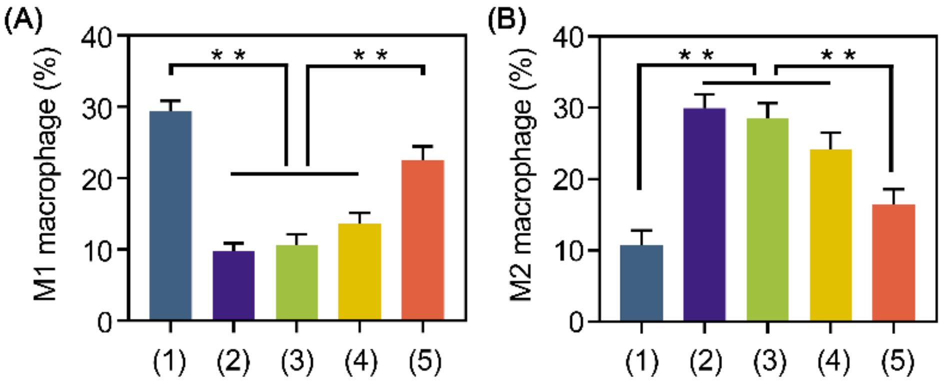

Figure S17. (A) Population ratios of M1 macrophages and (B) M2 macrophages were statistically quantified based on the flow cytometry results (**$p < 0.01$).

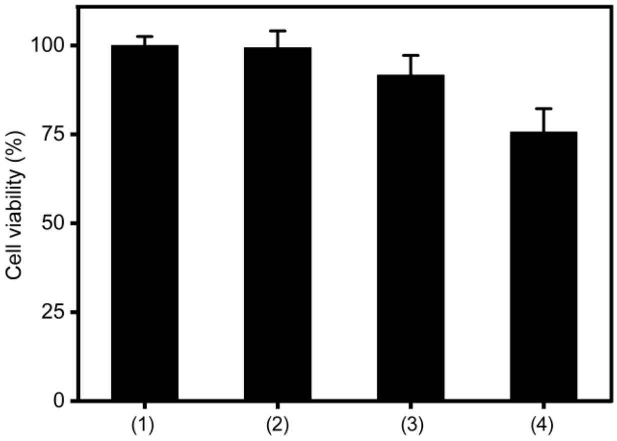

Figure S18. Cell cytotoxicity of macrophages with the treatment of BP nanosheets for 4 h. Afterwards, BP nanosheets were removed and macrophages were further incubated for another 8 h. Groups: (1) PBS, (2) Δ*T*, (3) BP nanosheets, (4) BP nanosheets + Δ*T*.

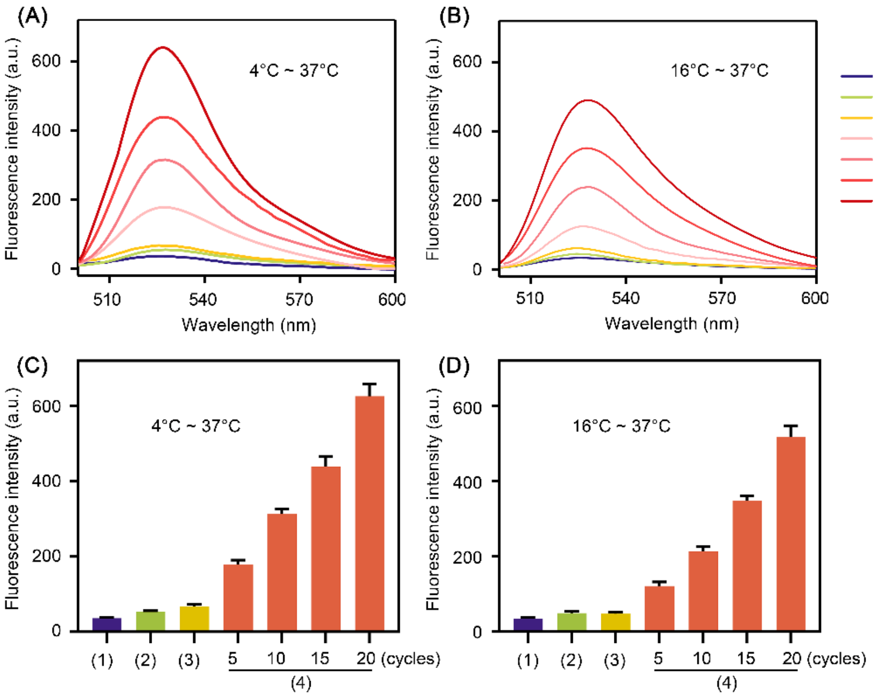

Figure S19. Fluorescence spectra of DCFH for ROS detection with a temperature variation of 4°C~37°C (A) and 16°C~37°C (B). (C–D) The maximum fluorescence intensity in different groups was compared based on the results of (A–B).

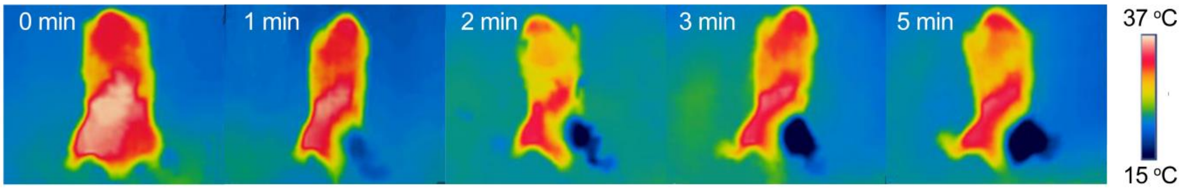

Figure S20. The thermal images of mice with different cold time.

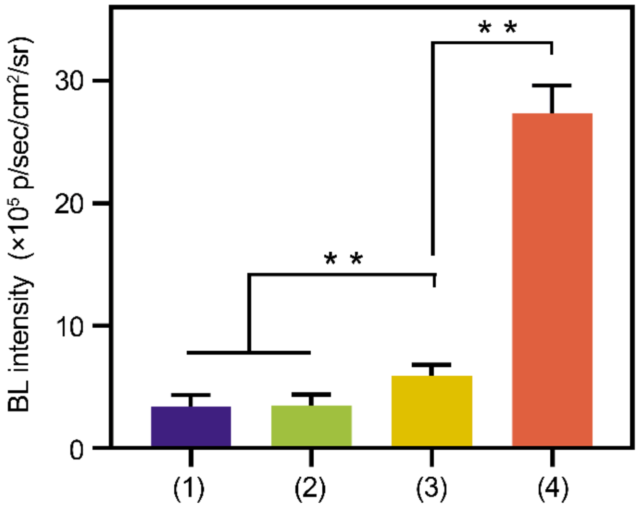

Figure S21. Intratumoral ROS was detected with L-012 as a bioluminescence probe. All groups: (1) blank, (2) Δ*T*, (3) BP nanosheets, (4) BP nanosheets + Δ*T*. $^{**}p < 0.01$.

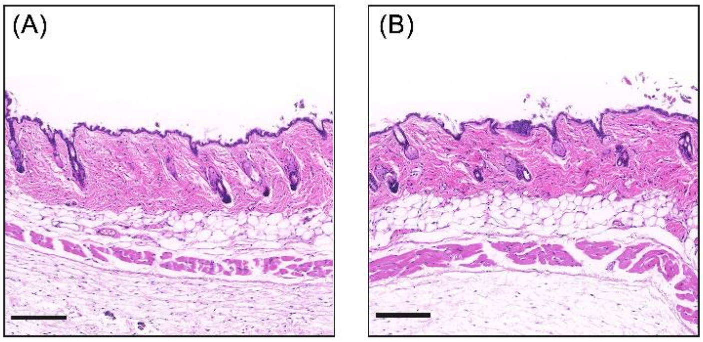

Figure S22. (A) H&E staining of mouse skin with and (B) without cold treatment (scale bar = 200 μm).

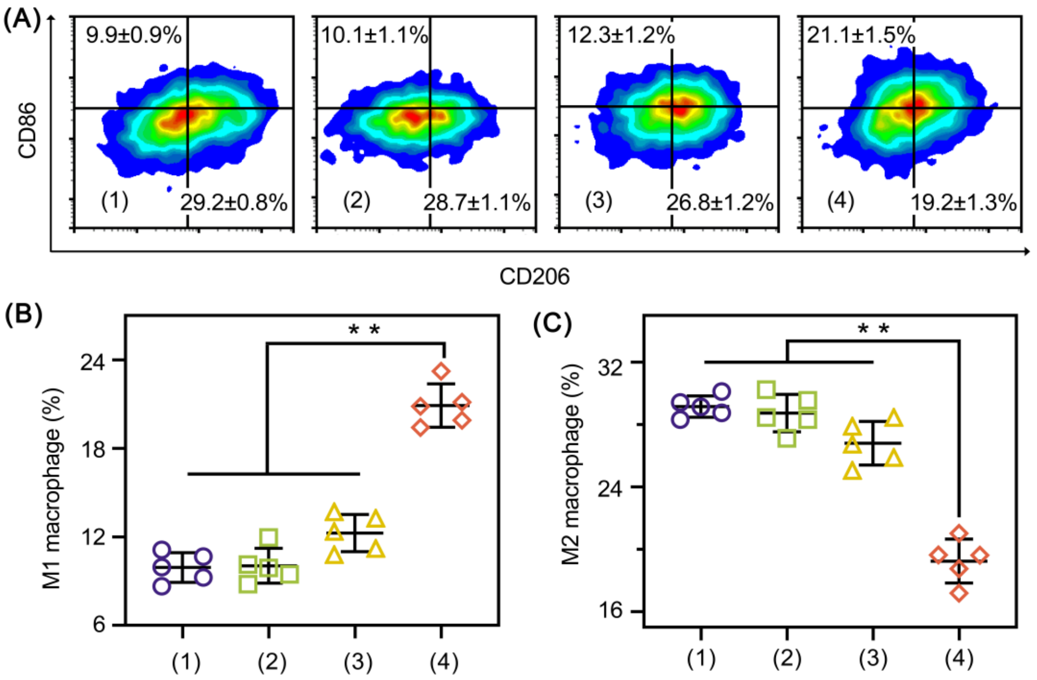

Figure S23. (A) Representative flow cytometry analysis of M1 (CD86^{high}CD206^{low}) and M2 macrophages (CD86^{low}CD206^{high}). (B) Population ratios of M1 macrophages and (C) M2 macrophages were statistically quantified based on the flow cytometry results (**$p < 0.01$). Groups: (1) PBS, (2) Δ*T*, (3) BP nanosheets, (4) BP nanosheets + Δ*T*.

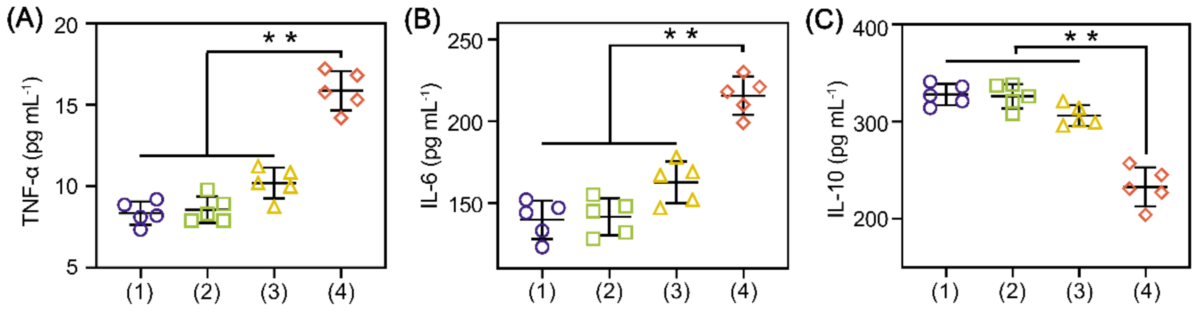

Figure S24. Serum cytokines (TNF-α, IL-6 and IL-10) were detected after different treatments. Groups: (1) PBS, (2) Δ*T*, (3) BP nanosheets, (4) BP nanosheets + Δ*T*. **$p < 0.01$.

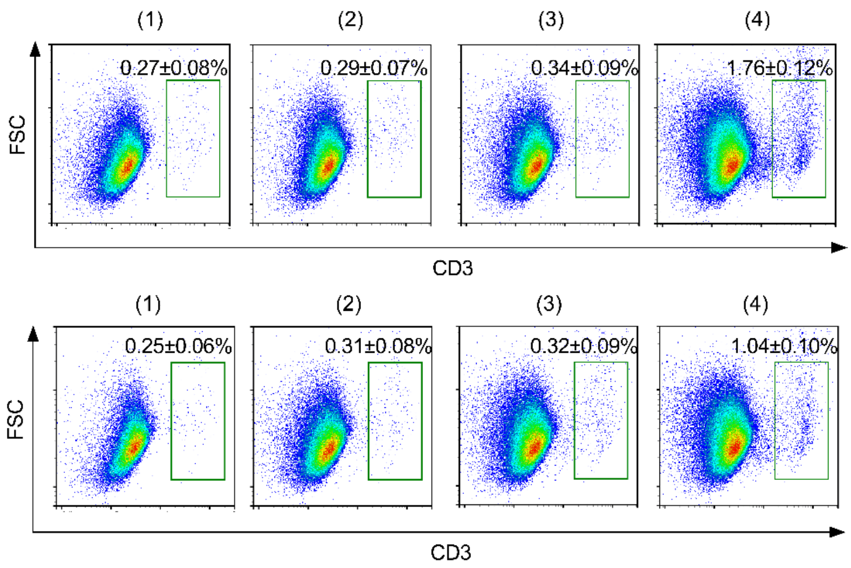

Figure S25. (A) The infiltration of T cells in the primary tumors and (B) distant tumors.

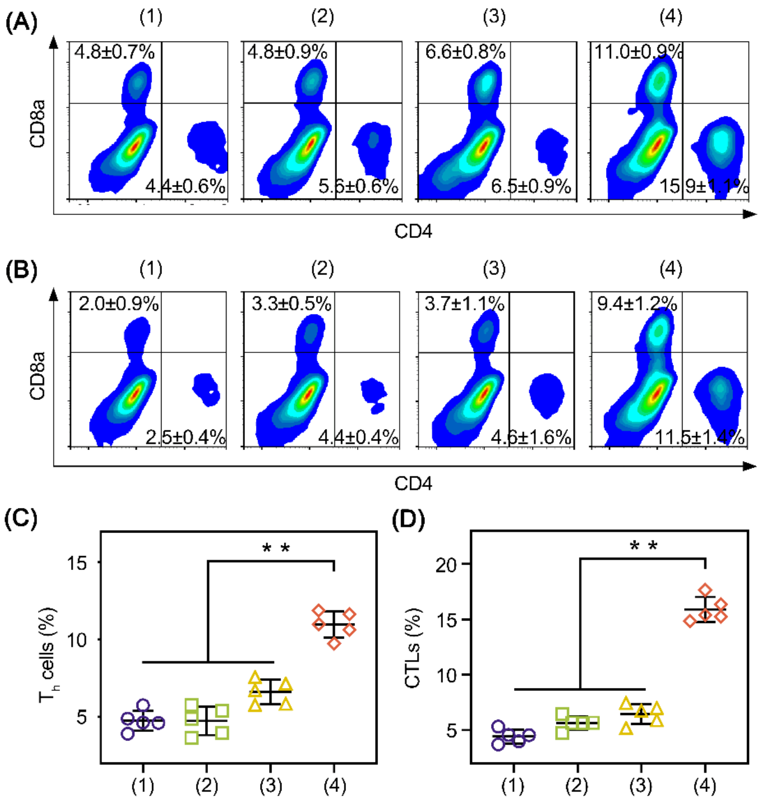

Figure S26. (A) Flow cytometry analysis of T cell activation in primary tumors and (B) distant tumors. (C) Fractions of tumor-infiltration T helper cells ($CD4^+$) and (D) cytotoxic T lymphocytes ($CD8^+$) in the primary tumors (gating on $CD3^+$). All groups: (1) PBS, (2) ΔT, (3) BP nanosheets, (4) BP nanosheets + ΔT. $^{**}p < 0.01$.

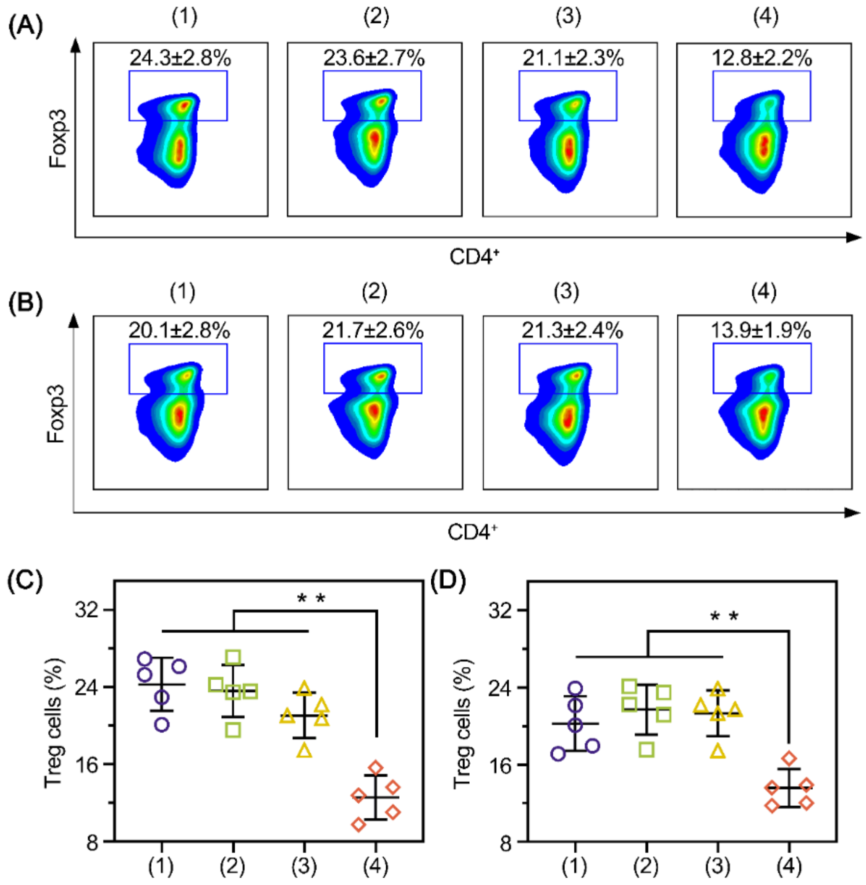

Figure S27. (A) Flow cytometric analysis of Treg cells in primary tumors and (B) distant tumors. The relative population of Treg cells ($Foxp3^+$) in (C) primary tumors and (D) distant tumors was quantified and compared. All groups: (1) PBS, (2) Δ*T*, (3) BP nanosheets, (4) BP nanosheets + Δ*T*. **$p < 0.01$.

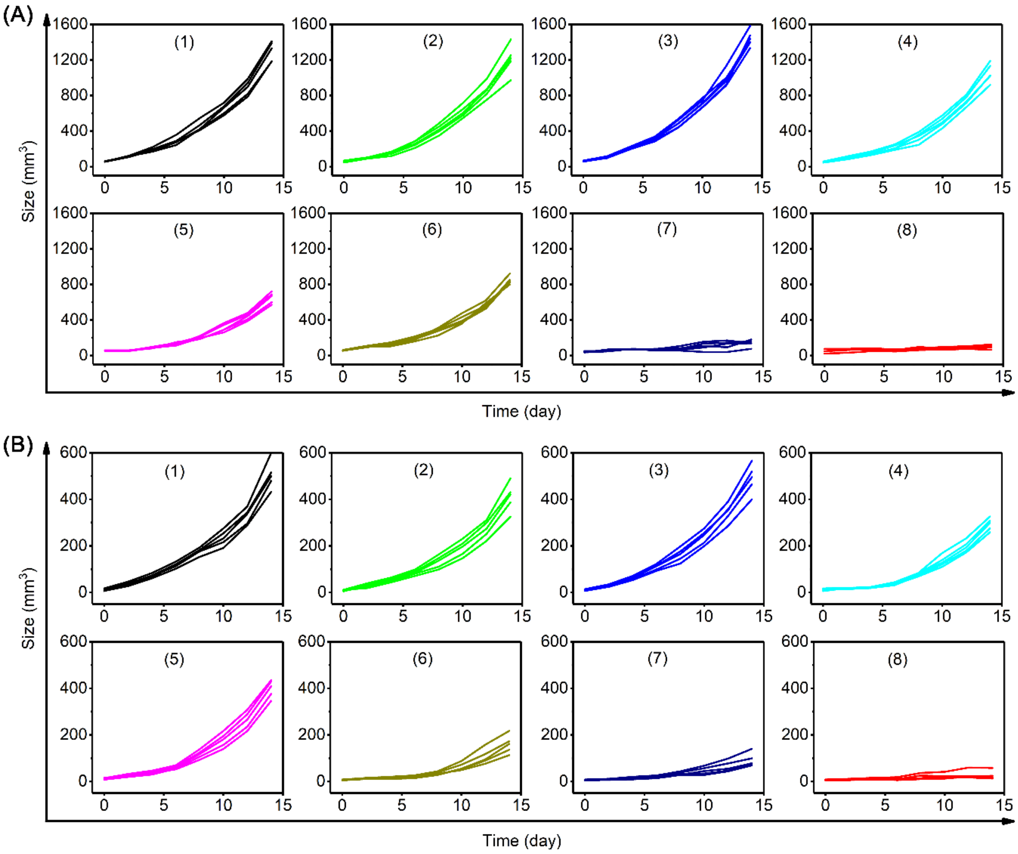

Figure S28. Individual growth curves of (A) primary and (B) distant tumors of mice after different treatments: (1) PBS, (2) αPD-1, (3) Δ*T*, (4) Δ*T* + αPD-1, (5) BP nanosheets, (6) BP nanosheets + αPD-1, (7) BP nanosheets + Δ*T*, (8) BP nanosheets + Δ*T* + αPD-1.

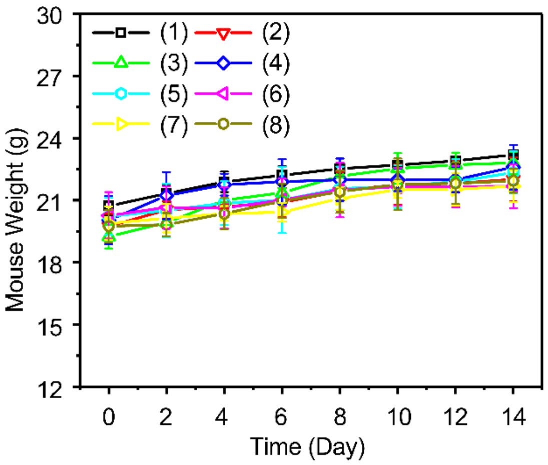

Figure S29. The change of mouse body weight over time in different groups: (1) PBS, (2) αPD-1, (3) Δ*T*, (4) Δ*T* + αPD-1, (5) BP nanosheets, (6) BP nanosheets + αPD-1, (7) BP nanosheets + Δ*T*, (8) BP nanosheets + Δ*T* + αPD-1.

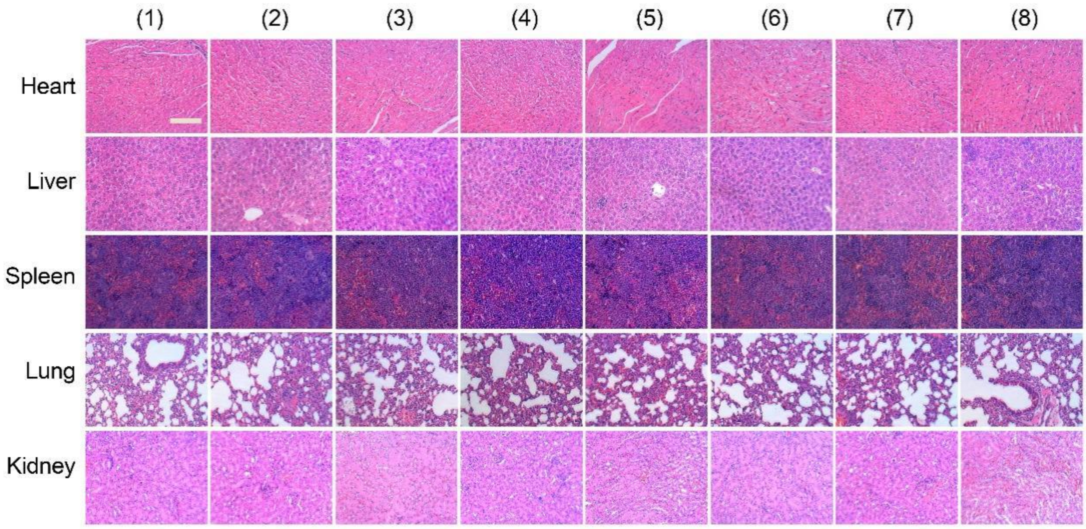

Figure S30. Main organs were histologically examined through H&E staining. All groups: (1) PBS, (2) αPD-1, (3) Δ*T*, (4) Δ*T* + αPD-1, (5) BP nanosheets, (6) BP nanosheets + αPD-1, (7) BP nanosheets + Δ*T*, (8) BP nanosheets + Δ*T* + αPD-1. Scale bar = 100 μm.

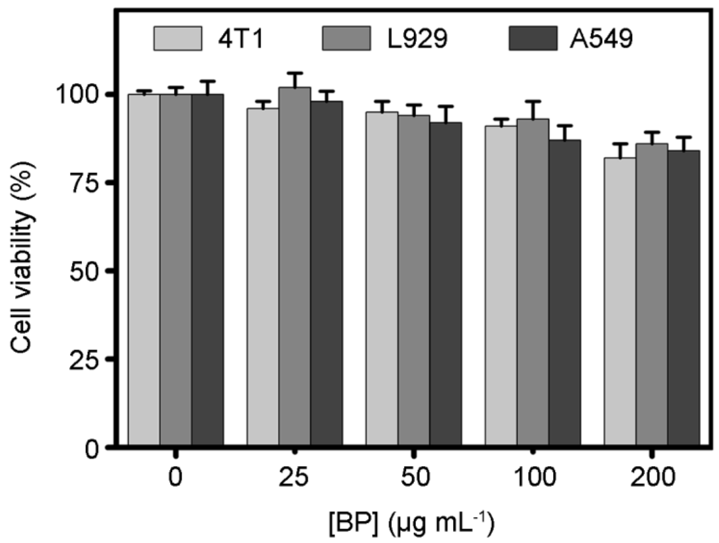

Figure S31. Cytotoxicity of BP nanosheets.

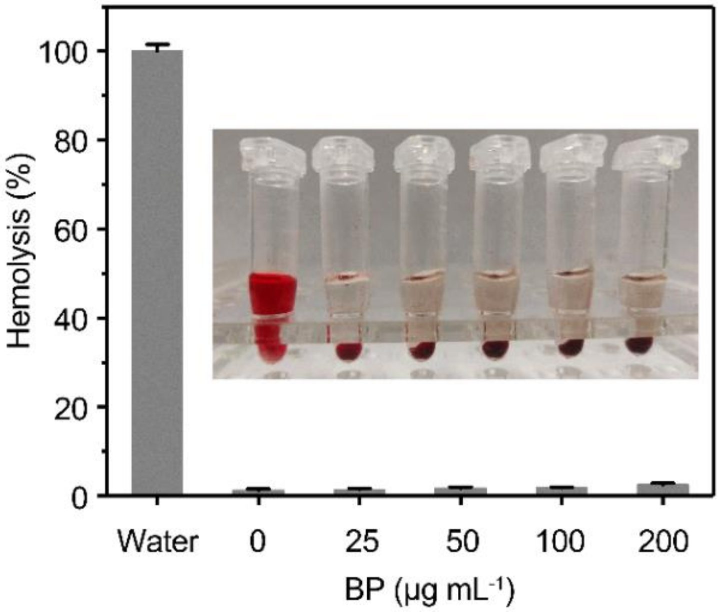

Figure S32. Hemolysis of erythrocytes at various concentrations of BP nanosheets.

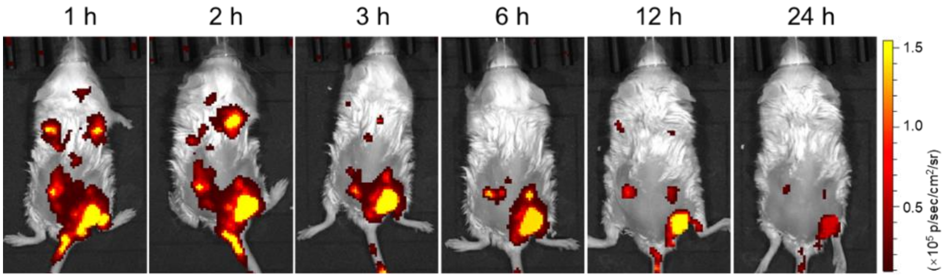

Figure S33. The *in vivo* biodistributions of Cy5.5-labeled BP nanosheets at different time points post intravenous injection.

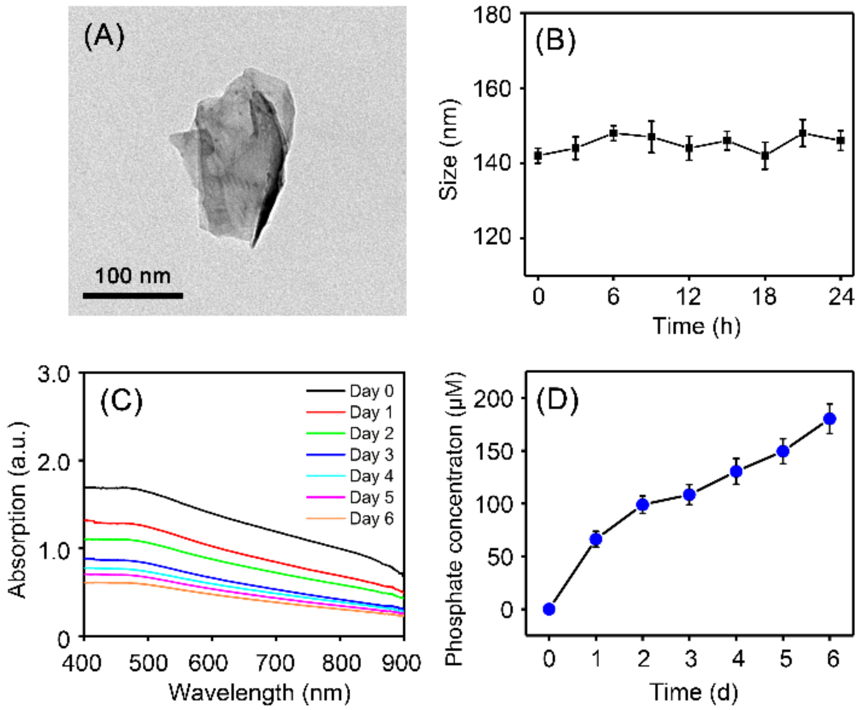

Figure S34. Stability of BP nanosheets. (A) TEM images of BP nanosheets in water for 24 h. (B) Dispersion stability of BP nanosheets in water was monitored for 24 h. (C) The UV-Vis spectra of BP nanosheets in distilled water with 6 days. (D) The phosphate concentration in the solution was gradually increased along with the degradation of BP nanosheets.

Notes and references

1 M. Xia, X. Wu, Y. Zhong, Z. Zhou, W-Y. Wong, *J. Mater. Chem., C.* 2019, ***7***, 2385.

2 X. Ding, G. Zhu, W. Geng, Q. Wang, Y. Wang, *Inorg. Chem.,* 2015, ***55***, 154.

3 A. Sazgarnia, N. Naghavi, H. Mehdizadeh, Z. Shahamat, *J. Therm. Biol.,* 2015, ***47***, 32.
